# Cryo-EM reveals the structural heterogeneity and conformational flexibility of multidrug efflux pumps MdtB and MdtF

**DOI:** 10.1128/mbio.02684-25

**Published:** 2025-12-10

**Authors:** Surekha Padmanaban, Clayton Fernando Rencilin, Rupam Biswas, Somnath Dutta

**Affiliations:** 1 Molecular Biophysics Unit, Indian Institute of Science 428779 , Bengaluru, India; 2 Department of Physiology and Cell Biology, The Ohio State University 167908 , Columbus, Ohio, USA; University of Cambridge, Cambridge, United Kingdom

**Keywords:** efflux pumps, MdtB, MdtF, membrane protein, detergent, *E. coli*, Cryo-EM

## Abstract

Resistance-nodulation-cell division (RND) efflux pumps are the major cause of multidrug resistance in bacteria, particularly in Gram-negative bacteria. They are complex molecular machines forming tripartite assemblies that actively transport out a wide range of antimicrobial agents, including antibiotics, biocides, and host defense molecules. However, the presence of multiple RND transporters with overlapping functions in a single bacterium raises questions about their individual functional relevance. In this study, we determined the cryo-electron microscopy (cryo-EM) structures of two distinct hydrophobic and amphiphilic efflux (HAE)-RND transporters from *Escherichia coli,* MdtB and MdtF. MdtB transporter is a part of the two-RND subunit system MdtABC. MdtF is a unique class of RND transporter whose expression is regulated by oxygen availability and is crucial for the survival of *E. coli* in anaerobic growth conditions. The cryo-EM structures of MdtB and MdtF reveal a novel conformational state of HAE-RND efflux pumps. While the MdtB structure adopts an intermediate state, MdtF displays structural dynamics in the presence of n-dodecyl-β-D-maltoside (DDM). MdtF at 2.8 Å resolution displayed a significant conformational change in the transmembrane core helices and flexibility in the transmembrane domain. Our findings highlight the significance of the novel structural state during the substrate transport mechanism. Furthermore, our structural analysis provides insights into drug-binding sites and the transport mechanism of these important transporters.

**IMPORTANCE:**

Resistance-nodulation-cell division (RND) efflux pumps are mainly responsible for multidrug resistance by extruding a wide range of antibiotics from bacterial cells. These pumps are frequently overexpressed in multidrug-resistant *Escherichia coli* strains, which are responsible for urinary tract infections and foodborne illnesses. In this current study, we resolved the structures of two hydrophobic and amphiphilic efflux (HAE)-RND transporters, MdtB and MdtF, using single-particle cryo-electron microscopy. Our study demonstrated novel structural states of MdtF during substrate transport. This knowledge provides valuable insights into the conformational transitions underlying substrate transport. Understanding these structural mechanisms fills a critical knowledge gap in the RND-mediated efflux process and lays the groundwork for structure-guided inhibitor design. Our findings contribute to ongoing efforts to develop novel therapeutic strategies to combat multidrug-resistant *E. coli* infections.

## INTRODUCTION

Multiple resistance-nodulation-cell division (RND) transporters are identified in Gram-negative bacteria that can transport a wide range of substrates. The RND family of transporters interacts with the periplasmic membrane fusion protein (MFP) and the outer membrane protein to form a macromolecular tripartite complex. The RND transporter uses proton motive force as an energy source to efflux substrates (1). RND transporters are classified based on the type of substrates they transport: hydrophobic and amphiphilic efflux (HAE subfamily) and heavy metal efflux (HME subfamily). *Escherichia coli* (*E. coli*) genome codes for six RND transporters in total; five efflux pumps belong to the HAE-RND family, and one efflux system belongs to the HME-RND family (1). The HAE-RND efflux pump of *E. coli* comprises AcrAB, AcrAD, AcrEF, MdtAB, and MdtEF (2–7). It transports a wide range of substrates like drugs, detergents, dyes, antibiotics, host defense molecules, bile acids, and organic compounds. The CusCFBA is the only HME-RND efflux system of *E. coli* that transports Cu and Ag (8, 9). Among the five HAE-RND transporters, AcrB, AcrD, AcrF, and MdtF fall into the Acr cluster (10). MdtB is the only HAE RND transporter from *E. coli* that belongs to the Mdt cluster (10). It has been reported that the substrate range for all the RND transporters is not very distinct; many RND transporters transport the same substrates. Many of these RND transporters have overlapping functions, while some RND transporters show distinct functions complementing the existing system. AcrB transports a wide range of substrates, while AcrD transports glycosides (6). Furthermore, growth condition plays a major role in regulating these RND transporter expressions and activity (3). However, very few RND transporters are structurally and functionally characterized (11).

We performed structural characterization of less explored efflux transporters MdtB from the Mdt class and MdtF from the Acr class. Although AcrB and MdtF are from the same class, both protein activation conditions are different (12, 13). They might have overlapping efflux activities, or their activities can complement in *E. coli*. MdtF transporter is 71% similar to *E. coli* AcrB, whereas MdtB is 28% similar to *E. coli* AcrB. Phylogenetic analysis has revealed that MdtF is closer to AcrB; both are from the Acr class of HAE-RND transporters. Whereas MdtB is from the Mdt class of HAE-RND transporters, it is closer to MdtC (10).

Among these, the AcrB transporter is extensively studied and acts as a benchmark for RND transporters. This transporter is ubiquitously expressed and reported to transport multiple substrates. MdtF from the RND family shows expression and activity in an anaerobic environment. It was reported that MdtF is upregulated more than 20-fold in anaerobic conditions (13). It actively transports toxic molecules produced in an anaerobic environment to survive extreme conditions (13–16). Anaerobically active RND efflux transporters are characterized by obligate anaerobes like *Bacteroides fragilis* (1). Facultative anaerobes like *E. coli* need a specific set of systems to activate and transport substrate in an anaerobic environment, apart from the commonly studied aerobic systems. *E. coli*, being a facultative anaerobe and opportunistic pathogen, is a good model organism to study. Additionally, this is found in the human gut and experiences an anaerobic environment. However, there is very little information we have about the RND transporters that coexist and function in both aerobic and anaerobic environments. It may be possible that AcrB and MdtF could show a functional crossover in *E. coli*.

On the other hand, MdtABC is a two-RND subunit system, which has two inner membrane RND transporters, MdtB and MdtC, coded in its operon (2, 4, 12). MdtBC is the only two-RND subunit system in *E. coli*. The two homologous RND transporter systems are identified in *Pseudomonas aeruginosa* (MuxABC), *Salmonella enterica* serovar Typhimurium, *Serratia marcescens*, *Erwinia amylovora*, *Pseudomonas putida*, and *Photorhabdus luminescens* (10). The two RND transporters, MdtB and MdtC, form an independent homo-trimeric assembly and hetero-trimeric oligomer. Interestingly, the heterotrimeric state MdtB2C is found to be active, whereas homotrimeric MdtB exists in an inactive form (12). The need for two RND transporters in a system and whether they work together or independently is still unclear. So far, the heterotrimeric RND transport mechanism has not been explored much.   

The structural architecture of the HAE-RND transporter is conserved. The HAE-RND transporter has three structural domains: docking domain (DD), porter domain (PD), and transmembrane domain (TMD) (17). The DD is the region of interaction with the MFP to form the tripartite complex. All the substrate channels lead to a single funnel/duct in the DD region, which connects the outer membrane channel of the tripartite system. Porter domain consists of four subdomains: PN1, PN2, PC1, and PC2 (17). These subdomains come together to form highly hydrophobic pockets. The subdomains form two binding pockets in porter domain—access binding pocket (ABP) and deep binding pocket (DBP). PC1 and PC2 form an ABP at the periphery of the protein, followed by PN1 and PN2 forming a DBP toward the core of the protein. The substrate binding pockets are rich in conserved hydrophobic aromatic amino acids. Gate loop (G-loop) divides these two pockets and acts as a switch/valve to close or open the path to DBP from ABP (18). The TMD has 12 transmembrane (TM) helices, and proton gradient-dependent conformational changes are translocated through the TMD (19).

So far, many conformational states of the RND transporters have been captured in its apo form and substrate-bound form. The asymmetric structure of AcrB was solved in the presence of substrates minocycline, doxorubicin, ethidium, dequalinium, detergents (n-dodecyl-β-D-maltoside [DDM], lauryl maltose neopentyl glycol [LMNG], and n-undecyl-beta-maltoside [UDM]) (18, 20–24), and inhibitors. The asymmetric structure of AcrB reveals that each protomer in the trimer exists in different conformational states, depicting the mechanism of substrate transport. The trimeric HAE-RND transporter undergoes conformational changes in an interdependent cyclic manner to transport the substrate. The protomers are in continuous cooperative movement during substrate translocation, known as a functional rotational mechanism (23). The three classical conformational states described by the HAE-RND transporters are the access state, the binding state, and the extrusion state in the order of the steps. The movement of the porter domain is responsible for the distinct conformational difference among these states. Opening and closing of PN1, PN2, PC1, and PC2 are observed in these states (17, 25).

In the access conformational state protomer, the protein is ready to take up the substrate from the peripheral opening-ABP and the vestibule formed by PC2 and the TM helix TM 8. It is observed that PC1 and PC2 moved away from each other to open (lose) the ABP. Following that, the protein experiences a binding state conformation, in which the substrate enters from the peripheral cavity to the DBP, passing the G-loop. Structurally, the subdomains PN1 and PN2 moved away to open the DBP cavity. The central helix from the neighboring extrusion monomer is inclined to block the exit gap in the DBP of the binding state protomer. Finally, in the extrusion state protomer, the substrate BPs and the channel entries are closed. At the same time, the funnel duct is opened because of the movement of the central helix to transport the substrate. The ABP and the DBPs are closed by the movement of PC1 and PC2 and PN1 and PN2. The TM8 is moved closer to PC2 in extrusion conformation to close the vestibule entry (17, 25). In this study, our target was to characterize the structure of two efflux transporters, MdtB and MdtF, from *E. coli*, which are involved in the extrusion of antibiotics, using single particle cryo-electron microscopy (cryo-EM) analysis. These two HAE-RND family transporters have been poorly characterized to date, and no structural information is currently available. In addition to elucidating their individual structures, we were interested in understanding how these transporters may interact or coordinate in the process of drug efflux. Thus, we employed cryo-EM-based structural analysis to elucidate the structure of efflux transporters, MdtB and MdtF.

## RESULTS

Preliminary computational studies of the MdtB and MdtF efflux transporters were carried out to gain insights into their sequence conservation and functional divergence, guiding subsequent structural analyses. To begin our analysis, we performed sequence alignments of efflux transporters MdtF and MdtB with transporter AcrB. MdtF shares 71% sequence identity with AcrB, whereas MdtB shares only 28% identity with AcrB, indicating that MdtF and AcrB are highly conserved, while MdtB is divergent (Fig. S1). We specifically analyzed conserved amino acid residues across the three transporters. Notably, Asp407, Asp408, and Lys938 (MdtF)—as well as Phe611—are conserved in all three proteins (Fig. S1A). Among these, the three charged residues (Asp407, Asp408, and Lys938), which are known to be essential for proton translocation, are conserved in MdtF and MdtB (Fig. S1A).

**Fig 1:**
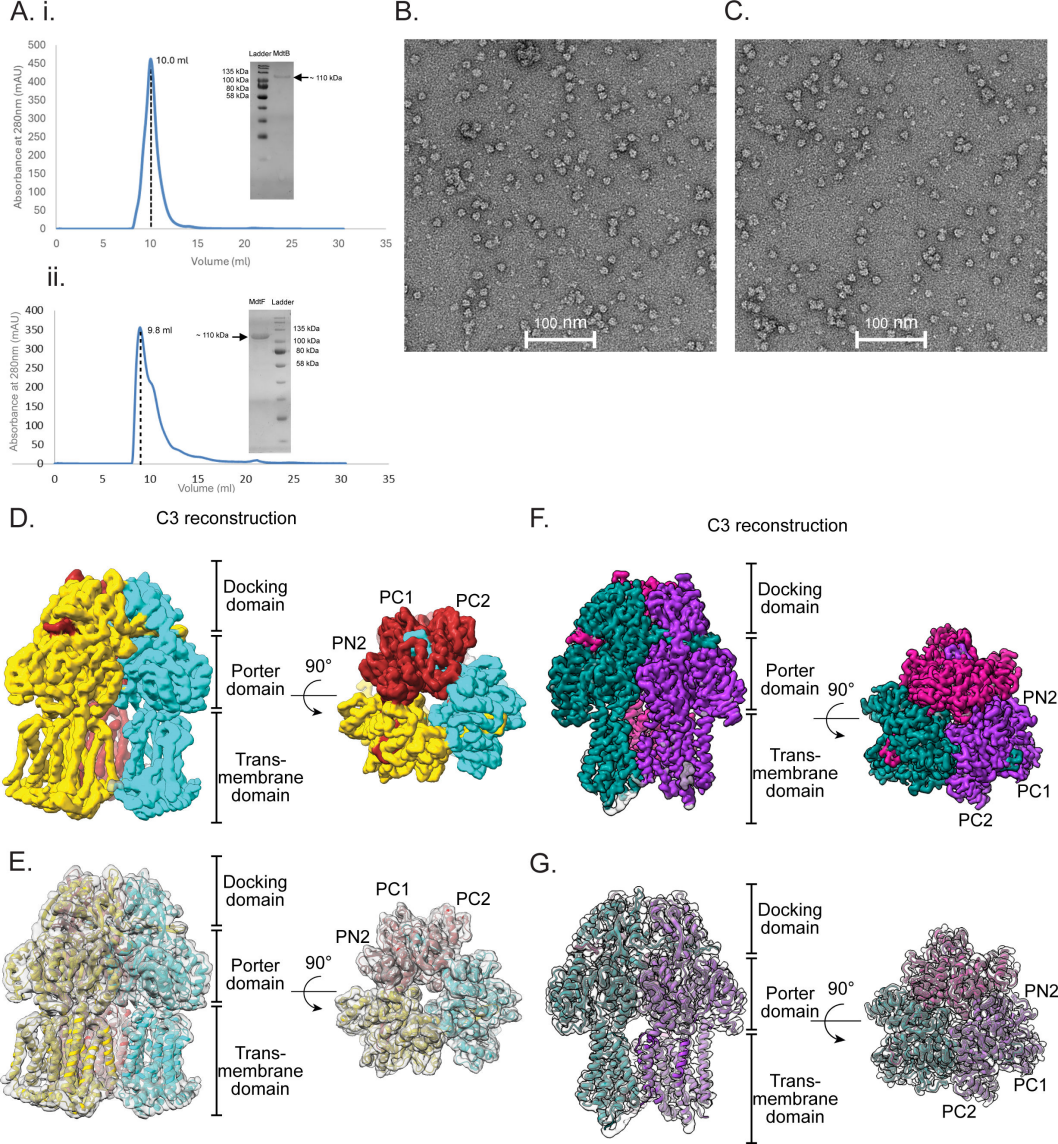
Purification and structural characterization of MdtB and MdtF. (**A**) (i) SEC elution of MdtB in the S200 column at 10 mL volume corresponding to the trimeric molecular weight of the protein. The eluted MdtB sample was analyzed using a 12% SDS-PAGE gel (inside). (ii) SEC elution of MdtF in the S200 column at 9.8 mL elution volume corresponding to the trimeric molecular weight and its SDS-PAGE of purified MdtF (inside). (**B**) Representative negative staining TEM (NSTEM) micrograph of MdtB. (**C**) Representative negative staining TEM (NSTEM) micrograph of MdtF sample. (**D**) Side view and top view of symmetric MdtB cryo-EM density map resolved with C3 symmetry at a global resolution of 3.9 Å. Each protomer is colored differently in the cryo-EM density. (**E**) Fitting of the built atomic model of MdtB in the cryo-EM density map. (**F**) Side view and top view of MdtF cryo-EM map resolved at a 2.8 Å resolution in C3 symmetry. Each protomer is colored differently in the cryo-EM density. (**G**) Fitting the built atomic model of MdtF in the cryo-EM map.

To facilitate structural studies, efflux transporters MdtF and MdtB were purified following detergent extraction with 2% DDM, with the concentration subsequently reduced to 0.03% for downstream purification. A two-step purification strategy was employed, beginning with immobilized metal affinity chromatography, followed by size exclusion chromatography (SEC). Both proteins eluted at approximately 9.8 mL on a Superdex 200 Increase column (Fig. 1Ai and ii), consistent with the trimeric form. SDS-PAGE analysis confirmed the purity and homogeneity of the samples, with a distinct band observed at ~110 kDa (Fig. 1Ai and ii). To further examine their oligomeric state, negative-stain transmission electron microscopy (TEM) was performed. The TEM micrographs revealed that both MdtF and MdtB assemble as homotrimers (Fig. 1B and C), in agreement with the SEC elution profiles. These results, together with the high expression yields and stable trimeric assemblies, provided a strong foundation for advancing to cryo-EM studies.

### Structural characterization of MdtB using single particle cryo-EM

Negative-stain TEM was initially employed to evaluate the structural integrity and cryo-EM suitability of the purified MdtF and MdtB proteins. The collected micrographs confirmed that both samples were monodispersed, ideal for cryo-EM-based structural study (Fig. 1B and C). Therefore, we proceeded to collect extensive cryo-EM data sets for both transporters to enable structural determination. The collected cryo-EM data were processed to generate reference-free two-dimensional (2D) class averages, which displayed characteristic top and side views consistent with HAE-RND family transporters (Fig. S2). Further, we performed a three-dimensional (3D) reconstruction of transporter MdtB, yielding a map at an overall resolution of ~3.9 Å at 0.143 Fourier shell correlation (FSC) (Fig. 1D; Fig. S3). This resolution was sufficient to confidently trace the polypeptide backbone and construct a reliable atomic model of MdtB (Fig. 1E).

**Fig 2:**
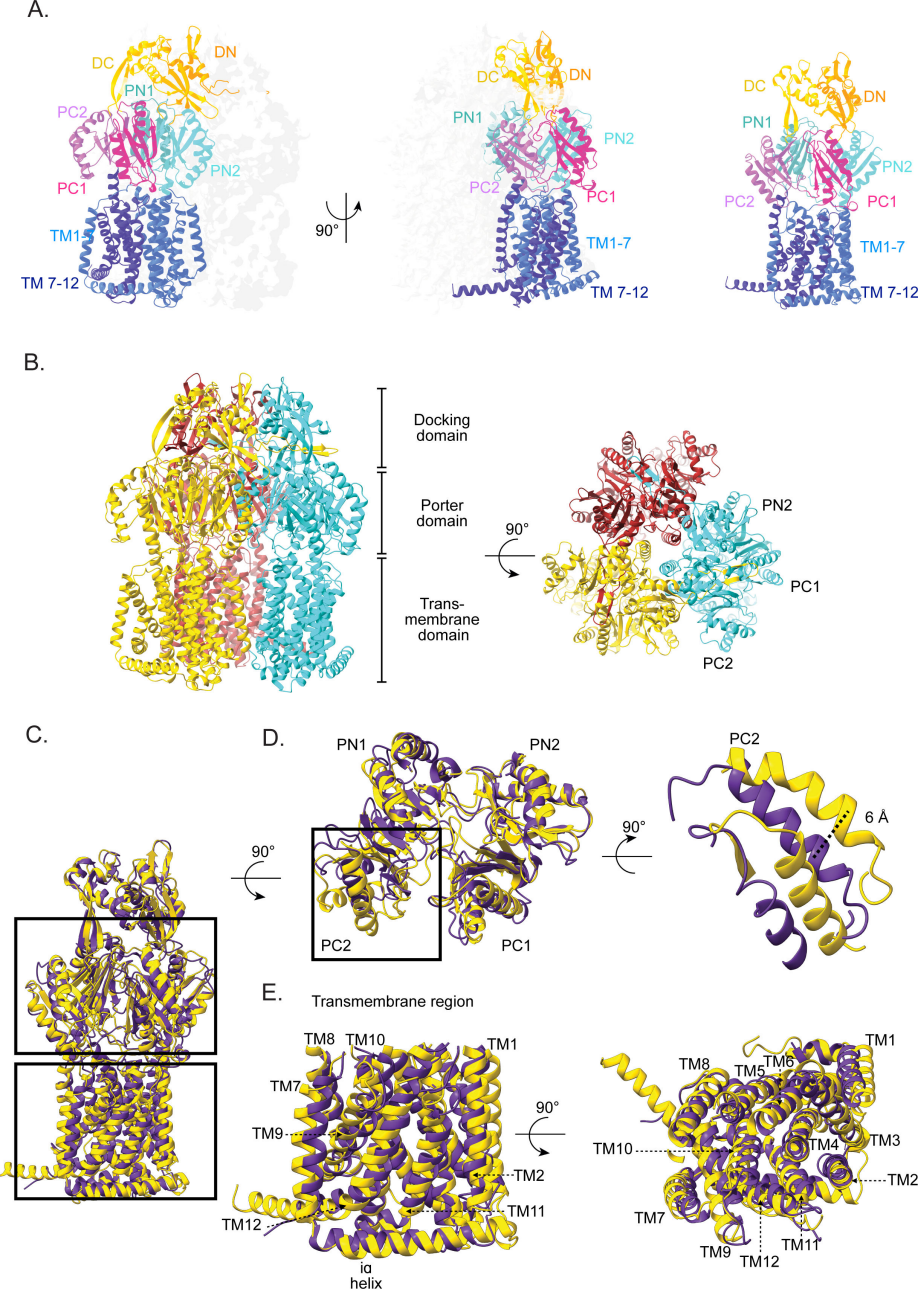
Atomic structure of MdtB and structural comparison with AcrB. (**A**) Monomeric model of MdtB monomer built from the cryo-EM density map. Subdomains are highlighted in varied colors and labeled. (**B**) The atomic model of the MdtB homo-trimer shows its tertiary architecture from the side and top. (**C**) MdtB and AcrB protomers (PDB ID: 2DR6- binding state) are superposed; yellow represents MdtB, while purple shows AcrB. (**D**) Top view showing the superposed image of the porter domain displaying a shift of the PC2 domain. The shift between the PC2 helix of MdtB and AcrB (PDB ID: 2DR6-binding state) is measured by calculating the center of mass shift between the two helices to quantify the movement. (**E**) Side view and top view of the superposed TMD.

We successfully resolved the first cryo-EM structure of homotrimeric MdtB, revealing an overall fold characteristic of the HAE subfamily within the RND transporter family. The reconstruction allowed us to clearly visualize all TM helices within each protomer, as well as the complete periplasmic domain. The homotrimeric assembly of MdtB closely resembles that of canonical RND transporters, such as AcrB from *E. coli* (Fig. 1D). Structurally, each protomer of MdtB comprises three major domains: the DD, the porter domain, and the TM domain (Fig. 1D). The DD, located in the periplasm, contains two subdomains—DN1 and DN2—that form a central funnel-like structure (Fig. 2A), which gathers substrates from multiple entry pathways and channels them outward via the tripartite efflux complex (Fig. 2A). The porter domain of MdtB is situated in the periplasmic region, in between the docking and TM domain, and consists of four subdomains: PC1, PC2, PN1, and PN2 (Fig. 2A and B). The fold of each domain is identical to the RND subfamily of proteins, and each has β-ɑ-β sandwich motif (Fig. 2A and B). These domains come together to form the substrate-BPs (Fig. 2A and B), helping RND transporters to recognize and extrude a wide range of antibiotics and toxic compounds.

To assess conformational variability within individual protomers of MdtB, we conducted an asymmetric reconstruction of protein MdtB. The resulting map showed that the overall trimeric architecture remained consistent. Superposition of the three protomers from the asymmetric reconstruction demonstrated a high degree of similarity, indicating minimal structural heterogeneity between the protomers. For further comparison, individual protomers of MdtB were aligned with the three known conformational states of AcrB protomers (Fig. S4B). Structural superposition of periplasmic domain revealed that the structure aligns most closely with the binding state of AcrB compared to access and extrusion states, with a main-chain root mean square deviation (RMSD) of 2.160 Å (Fig. S4B), indicating an overall similar but not identical conformation. Further, the change in the structural features was quantified by measuring the center of mass distance between subdomains PC1-PC2 and PN1-PN2 (Fig. S4C). Interestingly, this analysis revealed that the ABP in MdtB adopts a closed conformation. The structural features observed were distinct from the three states: access, binding, and extrusion. Specifically, the PC2 subdomain helix was found to be shifted approximately 6 Å compared to the binding state PC2, thereby narrowing the ABP (Fig. S4B; Fig. 2D). The conformation of the PN1 and PN2 subdomains in MdtB was closer to the binding state of RND transporters (Fig. S4B; Fig. 2D). However, the PN2 subdomain was displaced outward relative to PN1, resulting in the formation of a wider DBP (Fig. 2D). Additionally, the TMD of MdtB displayed a characteristic pseudo-two-fold symmetry, with TM helices 1-7 and 8-12 forming two structural repeats within the membrane-spanning region. The overall conformations of the TM helices and the arrangement of the core helices were largely consistent with those observed in the prototype AcrB structure (Fig. 2E).

### Cryo-EM analysis of MdtF shows the flexibility of the TMD

As outlined earlier, the trimeric structure of MdtF was initially evaluated using negative-stain TEM (Fig. 1C). To further characterize its structure at high resolution, we conducted cryo-EM analysis. A total of 4,000 movie frames were acquired, and 3D reconstruction applying C3 symmetry produced a map of MdtF at an overall resolution of approximately 2.8 Å, as determined by the gold-standard FSC at the 0.143 threshold (Fig. 1F; Fig. S5). This resolution permitted reliable model building, which was performed using *Phenix* and *Coot* (Fig. 1G). The resulting atomic model of MdtF revealed an overall fold characteristic of the HAE subfamily of RND transporters, including AcrB, MexB, AdeB, BpeB, and AcrD (Fig. 1F and G). The C3-symmetric density map resolved the core TM helices in all three protomers (Fig. 1F and G; Fig. S5). Notably, peripheral TM helices (TM7 and TM9 and TM10 and TM12), which interact with the surrounding DDM micelle, remained unresolved, likely due to flexibility.

**Fig 3:**
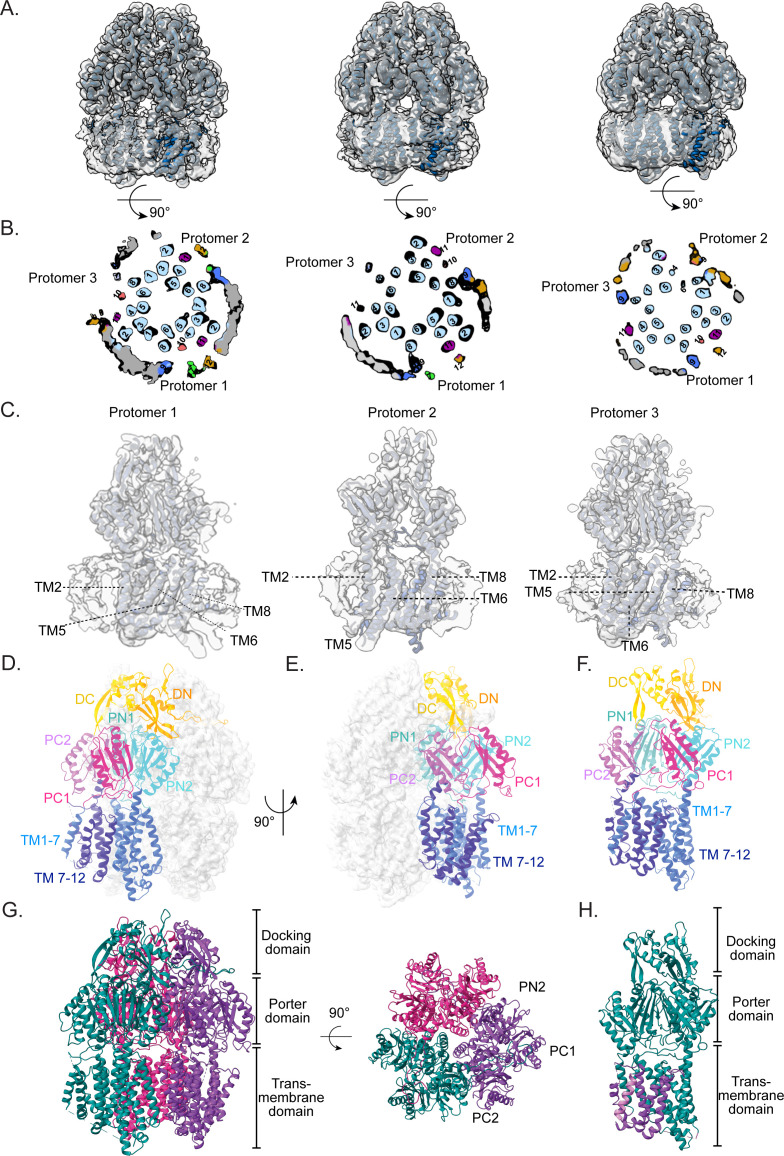
Asymmetric cryo-EM density maps display flexibility in the peripheral helices. (**A**) Three classes of MdtF solved with C1 symmetry are displayed. Rigid body fitting of built MdtF model in the refined 3D classes. (**B**) The cross-sections of the top view are shown for a better view of all the TM helices. Peripheral helices are highlighted in different colors for better understanding: TM9: blue; TM7: green; TM10: coral; TM11: purple; TM12: orange. In class 1 protomer 1, all 12 TM helices are resolved, while in protomers 2 and 3, some TM helices are missing. (**C**) MdtF fitting in class 1, where all the protomers are resolved. The posterior view of the fitting shows core helices are well resolved in all three protomers of MdtF class 1. (**D, E, F**) The monomeric model of MdtF was built from the asymmetric cryo-EM map. Each domain is highlighted in a variant color. (**G**) Side view and top view of the MdtF trimeric model. (**H**) Comparison of all three protomers by superposing. The flexible TM helices are highlighted in purple and magenta color.

Interestingly, asymmetric reconstruction of MdtF resolved the density corresponding to the peripheral TM helices, which were not observed in the C3 symmetry map. Upon 3D classification followed by refinement with C1 symmetry, the MdtF map displayed flexibility (Fig. 3A and B; Fig. S5). The central helices (TM1–6, TM8, and TM11) were consistently well resolved across all 3D classes analyzed (Fig. 3B). In contrast, the peripheral helices TM7, TM9, TM10, and TM12 are resolved variably in each class and protomer. Notably, while some peripheral helices, such as TM7, TM9, TM10, and TM12, could be resolved individually, they were never visualized concurrently in a single protomer. Remarkably, in class 1, protomer 1, all 12 TM helices were clearly resolved. However, the remaining two protomers of class 2 (protomer 2 and 3), TM7, TM9, and TM12 are not resolved, even after asymmetric reconstruction (Fig. 3B; Fig. S6). In class 2, protomer 1 displayed electron density for the helices TM7, TM9, and TM12, whereas these helices were absent in the other two protomers of class 2 (Fig. 3B). In class 3, TM9, TM10, and TM12 were resolved in protomer 1, while in other protomers, only TM9 and TM12 are resolved among the four peripheral helices (Fig. 3B). The variability observed in resolving the four peripheral TM helices across different protomers highlights the inherent flexibility within the TMD, which likely contributes to the difficulty in capturing all helices simultaneously in a single reconstruction (Fig. 3B). Additionally, local resolution analysis of the final cryo-EM map revealed that the peripheral helices were resolved at a lower resolution of approximately 6 Å (Fig. S5), suggesting increased flexibility of peripheral helices compared to the more stable core helices. Moreover, the traced TM helices exhibited substantial conformational deviations compared to other characterized RND transporters. These observations indicate that the MdtF exhibits different conformational behaviors, and not all 12 TM helices are resolved simultaneously in every protomer.

### Structure comparison between AcrB and MdtF

The MdtF trimeric model was built based on the completely resolved protomer (Fig. 3F). To analyze structural differences among RND transporters, we conducted a comparative analysis of MdtF with both AcrB and MdtB, revealing distinct variations across all major subdomains. Initially, we examined the conformational states of the porter domain by aligning the periplasmic region of the MdtF protomer with the three known conformational states of the *E. coli* AcrB protomer (Fig. S4A). This superposition revealed notable structural differences, which are discussed in the following sections.

**Fig 4:**
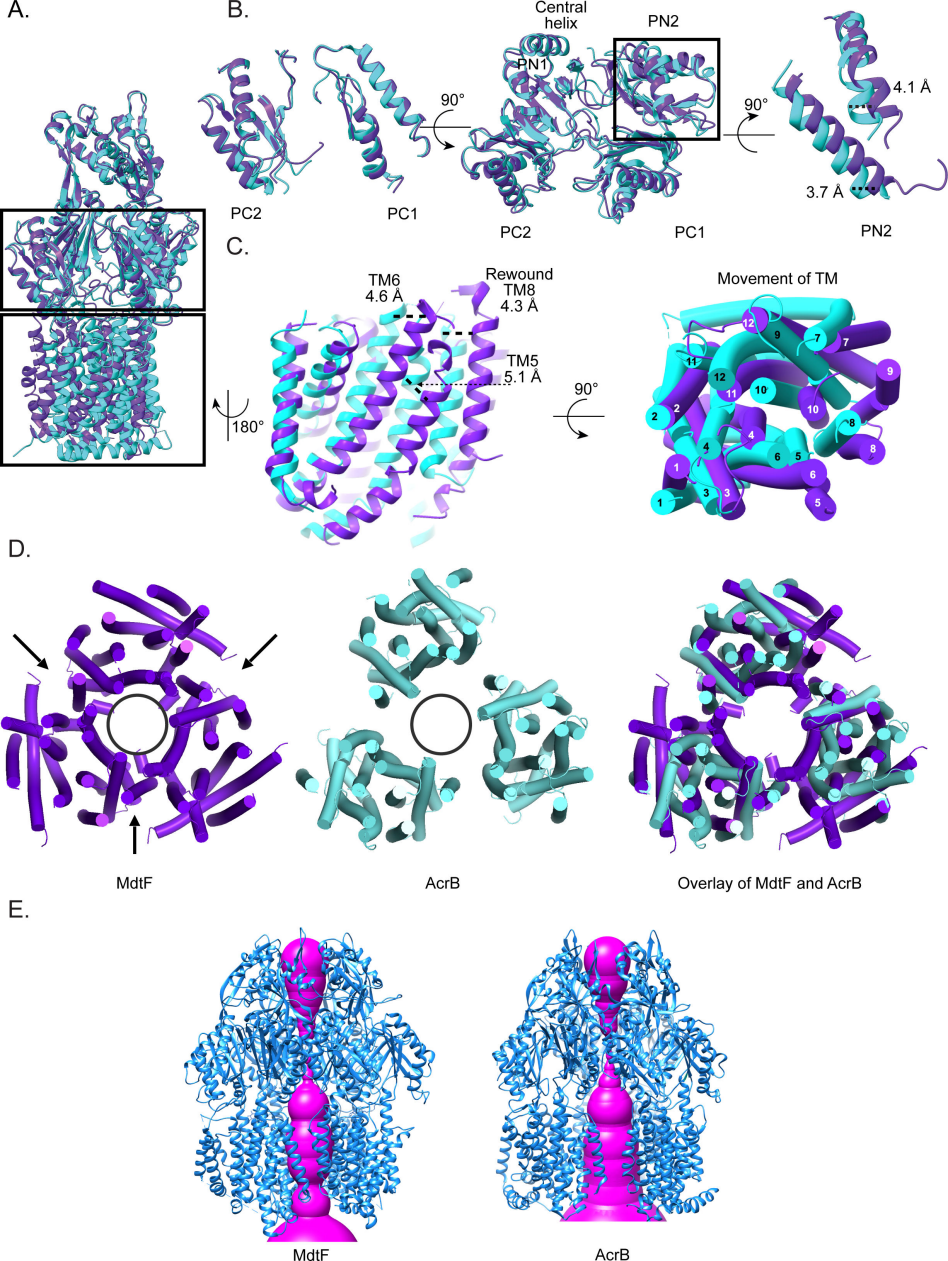
Structural comparison of MdtF with AcrB. (**A**) MdtF and AcrB monomers are superposed. (**B**) The periplasmic region is aligned with the binding state protomer of AcrB (PDBID: 2DR6). The periplasmic domain aligns well with the binding state protomer of AcrB. Minimal shift is observed in the PN2 helix, and the distance is calculated by measuring the local distance between residues. (**C**) Movement in the TM helices is highlighted. Core helices shift is measured by calculating the center of mass distance shift between the helices of MdtF and AcrB. The TM helices 5, 6, and 8 display a significant shift toward the pore. (**D**) Cross-sectional view from the top of MdtF and homotrimeric AcrB (PDB ID: 1IWG). The central pore of MdtF and AcrB was compared. Displacement of core helices in MdtF caused the central pore to close from the TM region. (**E**) Pore analysis is performed with the MOLE online web tool. Magenta highlights the central pore passage. Shrinkage of the pore size is observed in the bottom region of the MdtF.

AcrB is known to cycle through three distinct conformational states: the access, binding, and extrusion states. In our study, cryo-EM analysis yielded a single high-resolution structure of MdtF. To explore its conformational state, we compared the periplasmic domain of MdtF structure with the three protomer states of AcrB. The structural alignment and RMSD calculations were performed to identify the similarity between the periplasmic domains. The RMSD between MdtF and the binding-state AcrB periplasmic domain was approximately 1.01 Å, indicating the closest structural resemblance. In contrast, comparisons with the access and extrusion states yielded RMSD values of ~1.195 and ~1.789 Å, respectively (Fig. S4A). The structural changes observed in the porter domain were quantified by measuring the center of mass distance between subdomains PC1-PC2 and PN1-PN2 (Fig. S4C). These results suggest that the resolved MdtF structure most closely resembles the binding conformation of AcrB. Further analysis was made by superposing the MdtF structure with the binding state of AcrB (Fig. 4A). Although all the subdomains are highly similar to the binding-state protomer of AcrB, the PN2 helix in MdtF showed a local shift of approximately 3.7–4 Å compared to the PN2 helix of AcrB (binding-state protomer) (Fig. 4B). The inward movement of PN2 of MdtF appears to close the channel, resulting in a reduced DBP volume in MdtF, though it is wider than that observed in AcrB’s access state. Notably, all three MdtF protomers adopt a similar conformation, ensuring that the orientation of the central helices does not block the DBP exit of a neighboring protomer. These observations indicate that the porter domain of MdtF likely represents an intermediate state transitioning from the canonical binding conformation.

The high-resolution structure of MdtF offers valuable insights into its 3D architecture and highlights key structural differences from AcrB, particularly within the TM region. The most prominent variations were observed in the core helices of the TM domain. Consistent with prior reports describing a pseudo-twofold symmetry in the TM subdomains of RND transporters, our analysis revealed that MdtF also exhibits this architectural feature. Specifically, helices TM1–7 and TM8–12 form two symmetrical halves of the TMD (Fig. 3D and E). MdtF contains a total of 12 TM helices, TM 1–6 and TM8 were assembled as the core helices of the RND transporters, while TM7 and TM9–12 form the peripheral helix (Fig. 3G).

Structural superposition of MdtF with the binding-state conformation of AcrB revealed substantial displacements in several TM helices. A detailed comparison of the TM subdomain showed that helices TM4, TM5, TM6, and TM8 in MdtF were moved approximately 5 Å toward the central pore (Fig. 4C). The four helices were squeezed toward the center, while the remaining helices were twisted and moved away to facilitate the core helix movement. TM1, TM2, and TM3 of MdtF moved away from the center compared to AcrB. In previously characterized RND transporters, TM8 undergoes conformational changes, unwinding in the access state and rewinding in the extrusion state, to regulate vestibule opening for substrate entry. In MdtF, however, TM8 remains fully folded in an α-helical conformation and was angled in a manner that creates a widened vestibular space (Fig. S8D).

**Fig 5:**
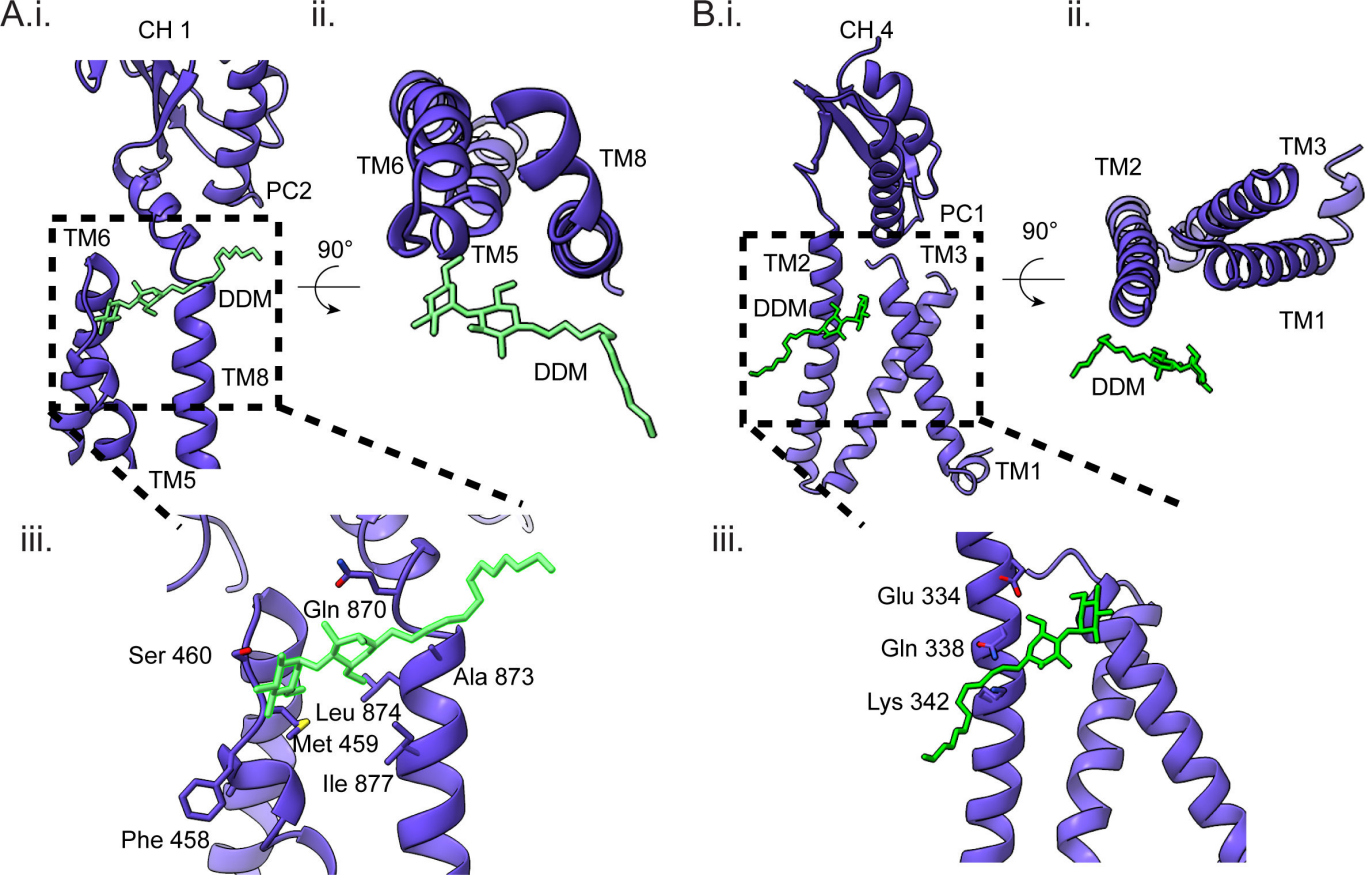
DDM bond in the CH1 and CH4 channel grooves. (**A**) (i) CH1 of the DDM entry site is shown. DDM bound near the TM8 vestibule entry. (ii) Top view of the DDM bound CH1 entry region. (iii) Amino acid residues surrounding the DDM-bound site are highlighted. (**B**) (i) CH4 of the DDM entry site is shown. DDM is bound near the TM1/TM2 groove of the MdtF. (ii) Top view of the DDM-bound CH4 entry region. (iii) Amino acid residues surrounding the DDM molecules are highlighted.

Furthermore, the cryo-EM map of MdtF revealed a well-defined density corresponding to DDM molecules within the TMD (Fig. S9). These molecules were located in specific BPs, in the groove between TM8 and TM6 and within the crevice formed by TM1 and TM2 (Fig. 5A and B). The modeled structure indicates that hydrogen bonding with polar residues in these regions stabilizes the DDM molecules, suggesting a potential role for detergent interactions in maintaining the structural integrity of the TM helices.

### Structural differences between the substrate BPs of AcrB, MdtF, and MdtB

Structural analysis of MdtB and MdtF revealed both adopted conformations distinct from the three classical states typically described for RND transporters. In RND transporters, substrates initially access the porter domain through the ABP, which was formed by the PC1 and PC2 subdomains. Subsequently, substrates were translocated toward the DBP, crossing the G-loop, which functions as a selective barrier. This switch or G-loop acts as a valve, controlling the entry of substrates into the DBP. The G-loop was inherently flexible due to the presence of glycine residues, and this flexibility is critical for the dynamic opening and closing of the entry channel, thereby regulating substrate passage into the DBP.

To further explore the structural basis of substrate gating, we examined the G-loop in both MdtF and MdtB. The G-loop in MdtF spans residues 611–621 (GGFGFSGQGQNN) and contains five glycine residues, one more than the corresponding loop in AcrB, suggesting enhanced flexibility (Fig. S1B). This increased flexibility likely facilitates the loop’s dynamic movement during substrate translocation. In the access state of RND transporters, the G-loop faces inward, blocking the DBP, whereas in the binding and extrusion states, it reorients away from the DBP, enabling substrate entry. Consistent with this, the G-loop in MdtF adopts a conformation that resembles the binding state, thereby exposing the DBP (Fig. S8B). In contrast, the G-loop in MdtB comprises residues 609–619 (VGVDGTNPSLN) and has a reduced glycine content, resulting in a more rigid conformation (Fig. S1B). In our structure, this loop appears displaced upward, further supporting its limited flexibility (Fig. S8B). Importantly, phenylalanine at position 615 is conserved across RND transporters, including MdtF, but is absent in MdtB (Fig. S1). Additionally, both BPs are surrounded by highly conserved aromatic residues, which are likely important for substrate recognition and stabilization.

Furthermore, we examined the structural and morphological features of the substrate transport channels in MdtB and MdtF using the MOLE and CASTp web tools (26, 27). The regulation of substrate entry at the vestibule is governed by the dynamic movements of the PC2 domain and the conformational change of TM8, which undergoes folding and unfolding transitions. These conformational changes were closely linked to the functional rotational mechanism characteristic of substrate transport in RND transporters. In the access state, TM8 unwinds at the interface between the periplasmic and TMDs and shifts away from PC2, thereby opening the entry pathway. In contrast, during the extrusion state, TM8 refolds into an α-helix, sealing the gap between PC2 and TM8 and effectively closing the channel.

**Fig 6:**
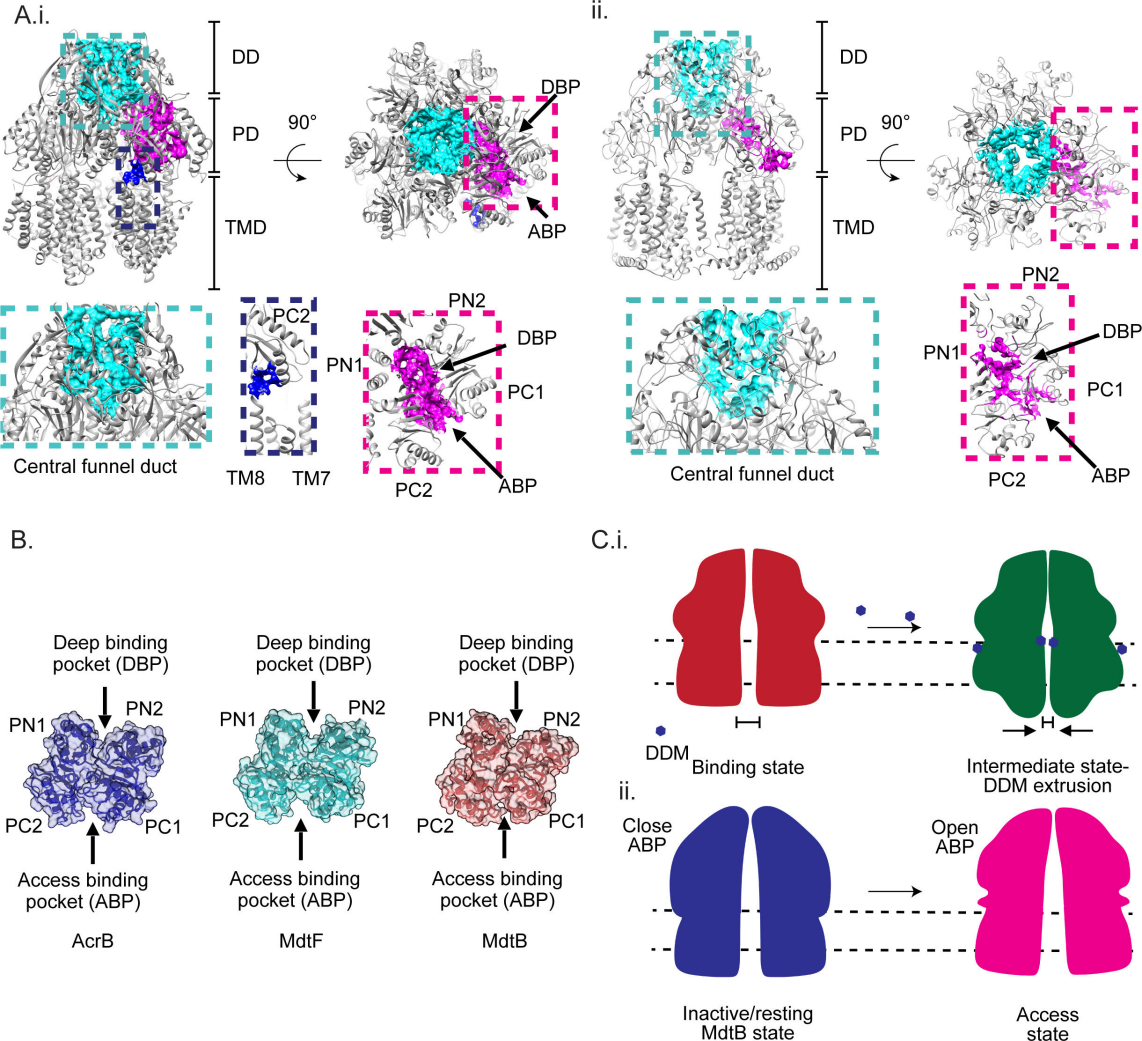
Comparison of BPs and substrate transport channels entries of MdtF and MdtB. (**A**) (i) Substrate transport channels of MdtF are highlighted in different colors. Access binding and DBP channels are wide open, and TM8 is in the rewound state with the vestibule opening. (ii) Substrate transport channels of MdtB are highlighted in different colors. DBP and narrower ABP are highlighted. (**B**) ABP and DBP of AcrB, MdtF, and MdtB are highlighted, and sizes are compared. MdtF ABP is wider compared to the other two proteins. The MdtB ABP is narrower than that of AcrB and MdtF. (**C**) (i) Schematic representation of MdtF intermediate structural state involved in the transport mechanism. Transition from the binding state of RND transporter to the solved intermediate state of MdtF upon DDM binding. (ii) Schematic representation of MdtB intermediate state in the RND transport mechanism. MdtB is in an inactive/resting state, then transitions to an active state.

In the case of MdtB, the TM8 helix appears refolded, effectively sealing the vestibule entry like the extrusion state. Additionally, the PC2 domain is shifted toward PC1 (Fig. 6Aii; Fig. S8D). In contrast, although the PC2 domain of MdtF adopts an open conformation leaving the ABP entry unobstructed (Fig. 6Ai; Fig. S8D). To better understand the implications of the observed conformational state, we analyzed the MdtF channel and found that the central pore is closed at the TM region (Fig. 4D and E). Specifically, the core TM helices (TM1 to TM6 and TM8) from each protomer are inclined inward and move toward the center. The inward displacement of TM helices creates a narrowed passage that appears to seal the central channel (Fig. 4D and E). As a result, the radius of the bottom opening in MdtF is reduced by approximately 6 Å compared to that of AcrB. These observations suggest that while MdtF periplasmic domain adopts a conformation closely resembling the substrate-binding state of AcrB, its TM closure likely hinders substrate transportation under this conformation. Extending this observation, structural superposition of MdtB and MdtF offered additional insights into their distinct substrate binding pocket configurations. The ABP in MdtB was narrower than in MdtF, due to an ~6 Å shift of the MdtB PC2 subdomain helix compared to the MdtF (Fig. S8C). In contrast, the DBP of MdtB was wider than that of MdtF, with the PN2 domain shifted away from PN1. In the apo form of MdtB, both the vestibule and ABP were closed, while the DBP remained open. On the other hand, MdtF displayed a wider ABP due to the outward displacement of the PC1 and PC2 domains. The ABP in MdtF was broader than in the access-state conformation of other RND transporters and closely resembled the binding-state configuration (Fig. 4B and 6B).

## DISCUSSION

In this current study, we employed cryo-EM-based structural analyses to characterize the efflux transporters MdtB and MdtF under the superfamily of HAE-RND transporters from *E. coli*. Multiple sequence alignment of HAE-RND transporter displayed a low percentage of sequence identity between MdtB and AcrB (28%), while a high percentage of sequence identity is observed between MdtF and AcrB at 71% (Fig. S1A and C). Moreover, based on the phylogenetic analysis, MdtB and MdtF belong to two different clusters, Mdt and Acr, respectively (10). Among the five HAE-RND transporters, AcrB, AcrD, AcrF, and MdtF fall into the Acr cluster (10). MdtB is the only HAE RND transporter from *E. coli* that belongs to the Mdt cluster (10).

We performed structural characterization of less-explored efflux transporters MdtB from the Mdt class and MdtF from the Acr class. Our findings reveal that these RND transporters were adopted a structurally distinct state. The solved MdtF structure is a symmetric trimer; upon performing a structural comparison with other RND transporters, we observed that the MdtF structural state was close to the binding state of AcrB (PDB ID: 2DR6). The periplasmic domain structural features resemble the binding state (T state) with open ABP, wider DBP, and G-loop facing outside. Also, the conformation of the G-loop resembled the binding state protomer, which is outward-facing.

Interestingly, the TMD structural state is very different from that of the reported RND transporter. In the cryo-EM map of MdtF, it was also observed that the three peripheral helices were solved at low resolution in an asymmetric protomer and couldn’t capture all the peripheral helices (TM7, TM9, TM10, and TM12) when symmetry was applied (Fig. 3). Asymmetric reconstruction demonstrates the flexibility of TM domains of individual protomers within MdtF, where all 12 TM helices were captured in protomer 1 and nine TM helices in protomers 2 and 3 (disordered TM7, TM9, TM10, and TM12) of MdtF (Fig. 3). These observations suggested that the peripheral helices were flexible, which could be challenging to capture in the structure. Since the protein was in its native environment and was in constant movement before freezing, it was not in a static state. Therefore, heterogeneity in the sample was best captured using the cryo-EM technique. The discrete heterogeneity in the sample could be captured by performing 3D classification, which clusters the discrete groups of samples from a data set in different classes. Whereas, if the heterogeneity in the sample is continuous, it is hard to capture by clustering into groups. The structure comparison shows that core TM helices TM 4, 5, 6, and 8 were moved toward the central pore, causing the pore to close. The TM helices of MdtF showed a major shift in the position in cryo-EM structure, and the TM helices were displaced ~4.1 and 5.5 Å toward the center. Many such transient conformations of RND transporters have been solved using the cryo-EM technique in the last decade. In the AdeB structure, intermediate states of the transport mechanism were solved using a cryo-EM technique, where the opening of the TMD was observed (28). We observed a similar flexibility behavior of the MdtF map when processing the data collected using a Talos Arctica 200 kV microscope (Fig. S7).

In RND transporters, four channels are identified to transport different types of substrates (CH1–CH4) (19, 23). Among the four channels, three of the channel entries are from the TMD. The channel 1 (CH1) entrance is located at the vestibule between TM8 and PC2, opening to the cell membrane. Meanwhile, the CH4 entrance is between the TM1/TM2 cleft. Both have common substrates, such as β-lactam, fusidic acid, and DDM. The CH3 entrance faces the central pore, which is specific to small polar aromatic molecules, and the CH2 opening is via ABP. Although substrates take up different channel entrances, they pass through ABP and DBP. Sometimes, it bypasses one of the binding pockets. We noticed that two DDM molecules are bound in our structure, one near the CH1 entrance (between TM8 and TM6) (Fig. 5A) and the other binding site at the CH4 entrance (groove between TM1 and TM2) (Fig. 5B). In an earlier study, it was shown that fusidic acid binds to the deep TMD-BP and then allosterically regulates the binding of substrates in CH1 and CH4 binding pockets. During this process, movement in TM11/TM12 was observed in the binding state protomer on substrate binding, creating an ~1700 Å^3^ cavity to accommodate fusidic acid (FUA); a similar cavity was created in our structure locally (23).

In this current cryo-EM structure, DDM was bound to all the binding pockets in TMD except the TMD-BP (surrounded by TM2, TM4, and TM10–12). It was bound at the same positions as of DDM and fusidic acid in the TFI-LIG1 state (6zod) of the allosteric transport mechanism (Fig. 5A and B). The proposed transport mechanism of DDM considers only the CH1 or CH4 channels at a time (18, 20, 22–24). In our findings, we observed that the DDM was bound simultaneously to all the sites, similar to the allosteric binding of FUA in the TMD. We speculate that, upon saturating the TM binding domains with DDM, the conformational transition happens in the TMD and will extrude the substrate to the periplasmic domain by squeezing/shrinking the TMD. Similar to proton translocation or allosteric binding in TMD-BP, the conformational change has been induced from the TMD to the periplasmic domain. As a result, the TMD experiences extrusion on saturation of the BP while the periplasmic domain is still in the binding state (Fig. 6Ci). In addition, the binding of multiple DDM at different BPs made us believe that the structure is dynamic in the TM region because of the DDM binding and extrusion process. We hypothesize that the solved structural state was the post-extrusion state of MdtF, where the central pore was closed post-transportation of the DDM molecule.

A recent preprint (29) has reported the structure of MdtF in its native lipid environment. The structure provides additional complementary information to support the structural changes we observe in MdtF in the presence of DDM. In the native lipid environment, MdtF aligns closely with the AcrB structure and does not exhibit notable conformational change or flexibility in the TMD of MdtF. Whereas our MdtF structure was determined in the presence of DDM, which appears to induce conformational changes and increase flexibility in the TMD of MdtF. These observations are consistent with our proposed intermediate state of MdtF, which is attributed to DDM binding and its subsequent extrusion.

Structural analysis of MdtB revealed a periplasmic domain conformation distinct from any previously reported structural states of RND transporters. RND transporters typically contain four channels, all of which were examined in MdtB. In the case of CH1, the TM8 helix adopts a refolded α-helical configuration, effectively sealing the vestibule entry similar to the extrusion state (Fig. S8D). Although this TM8 conformation resembled the extrusion state, the positions of TM8 displayed a significant shift compared to refolded TM8 of MdtF and AcrB (Fig. S8D). Similarly, the CH2 entry from the ABP was closed because of the narrower ABP and an expanded DBP (Fig. 6Aii and B). These results led us to hypothesize that the solved structure of MdtB represented an inactive or resting conformation that likely preceded the access state—the state where the BPs open subsequently to allow substrate entry (Fig. 6Cii). A key structural element, the G-loop—critical for substrate binding and selection—appears shorter and more rigid in MdtB compared to MdtB (Fig. S8B). This rigidity was because of the reduced number of glycine residues: MdtB contains only two glycines within its G-loop, whereas typical RND transporters possess four, and MdtF contains five, enhancing its flexibility. Furthermore, MdtB lacks the conserved GFGF motif and does not have a phenylalanine residue within the loop—specifically, the highly conserved F615, known to be essential for substrate transport in RND transporters. Instead, the G-loop of MdtB is enriched in polar aliphatic residues and is structurally displaced upward, in contrast to other RND transporters, where the G-loop acts as a dynamic valve between the ABP and DBP. These observations suggest that the G-loop of MdtB may play a limited role in substrate recognition and selection. To further explore this unusual feature, we examined the G-loop of MdtC, the bicomponent partner of MdtB. The MdtC loop is four residues shorter and similarly lacks aromatic amino acids (Fig. S1A). These structural characteristics, shared by both MdtB and MdtC, suggest that the bicomponent RND system possesses an unconventional G-loop. This may lead to altered substrate specificity or facilitate distinct transport mechanisms depending on whether the transporter operates in a homotrimeric or heterotrimeric state.

The asymmetric structures of RND transporters have provided critical insights into the functional rotational mechanism underlying substrate efflux, as demonstrated in AcrB bound to substrates such as doxycycline, fusidic acid, macrolides, erythromycin, and levofloxacin (20, 25, 30–32). In addition to the canonical access, binding, and extrusion states, numerous alternative conformational states have been resolved that do not align with this classical model (23, 28, 33). Cryo-EM technique was used to capture these non-canonical conformations, including resting and intermediate states of HAE-RND transporters (28, 34). The resting conformation, in particular, is described as an inactive state prior to channel opening and substrate entry (pre-access state). Furthermore, several intermediate states display a combination of structural features from the three traditional states, which have also been reported; one such example is BpeB and BpeF (33). These findings highlight the continuous structural rearrangements that occur during substrate translocation, making it inherently difficult to capture the full spectrum of dynamic states experimentally. Although various drug-bound structures have been resolved, the complete sequence of conformational transitions remains poorly understood. Molecular dynamics simulations of AcrB results have supported this idea that highlighted the intermediate states and demonstrated the mobility of TM helices (35). These studies suggested that RND transporters are intrinsically dynamic, adopting a broad range of intermediate conformations. Our study also highlights the dynamic behavior of RND transporters under defined experimental conditions by capturing transient intermediate states of MdtF and MdtB.

In summary, this study offers significant insights into the structural dynamics of RND efflux transporter, with a focus on MdtF and MdtB from *E. coli*. Importantly, we resolved previously undefined conformational states of both MdtF and MdtB, revealing novel structural features. Despite MdtF sharing high sequence similarity with AcrB and belonging to the same Acr cluster within the HAE-RND family, its structure is different from that of AcrB. In contrast, MdtB, which belongs to a different RND cluster, shows a high structural resemblance to AcrB. MdtF displays notable flexibility within its TMD, whereas MdtB features a more rigid and stable TM architecture, similar to other well-characterized RND transporters such as AcrB, MtrD, AdeB, and AdeJ. Our comparative analysis highlights that these structurally divergent RND transporters employ distinct mechanisms for substrate recognition and transport.

## MATERIALS AND METHODS

### Multiple sequence alignment

Multiple sequence alignments of RND transporters were performed using ESPript 3.0 and Unipro UGENE online tools. Consensus residues were highlighted, and the imported conserved residues of RND transporters were marked.

### Strains and constructs

The MdtF and MdtB genes were PCR amplified from the *E. coli* genomic DNA, and they were cloned into the pET21a (+) vector. It was cloned between the NdeI and XhoI restriction sites with the C-terminal octa-His tag. *E. coli* TOP10 cells were used for cloning and purification of the plasmid. For MdtF overexpression, *E. coli BL21ΔacrB acrD mdtABC* (NKE1317) cells were used; these cells were generously provided by Dr. Kunihiko Nishino (Institute of Scientific and Industrial Research, Osaka University). Kanamycin (50 µg/mL) and ampicillin (100 µg/mL) were used as selection markers for the cells and MdtF, respectively.

### Protein purification

To express the wild-type efflux transporters of MdtB and MdtF, *E. coli BL21ΔacrB acrD mdtABC* (NKE1317) cells carrying the appropriate plasmid were grown in Terrific Broth. When the optical density reached 1.0–1.5, cells were induced with 1 mM IPTG and grown at 20°C for 18 h. The harvested cells were pelleted by centrifugation and resuspended with lysis buffer, 50 mM Tris pH 8.0, 500 mM NaCl, 500 µM EDTA, 0.2 mg/mL (wt/vol) lysozyme, and 1 mM phenylmethylsulfonyl fluoride (PMSF). The sample was lysed by sonication for 1 h at 30% amplitude with 5 s on and 10 s off condition. After lysing the cells by sonication, cell debris was removed by centrifugation (8,000 rpm, 4°C, 20 min), and the supernatant was subjected to ultra-centrifugation (30,000 rpm, 4°C, 2 h) to separate the total membrane. The membrane pellet was solubilized in membrane resuspension buffer, 50 mM Tris pH 8.0, 500 mM NaCl, 5 mM imidazole, 10% (vol/vol) glycerol, 2% (wt/vol) DDM, for overnight at 4°C. The remaining insoluble pellet fraction was separated by ultracentrifugation (30,000 rpm, 4°C, 30 min). The supernatant was incubated with pre-equilibrated Ni-NTA agarose beads (Qiagen NI-NTA agarose beads) with the binding buffer, 50 mM Tris pH 8.0, 500 mM NaCl, 5 mM imidazole, 10% (vol/vol) glycerol, 0.03% (wt/vol) DDM (buffer A). The Ni-NTA beads were washed with wash buffer, 50 mM Tris pH 8.0, 500 mM NaCl, 50 mM imidazole, 10% (vol/vol) glycerol, 0.03% (wt/vol) DDM (buffer B), and the proteins were eluted with elution buffer 50 mM Tris pH 8.0, 500 mM NaCl, 300 mM imidazole, 10% (vol/vol) glycerol, 0.03% (wt/vol) DDM (buffer C). The eluted protein is concentrated with a 100 kDa molecular weight filter Amicon spin column concentrator. The protein was purified further using SEC column Superdex 200 10/300 Gl with the SEC buffer, 25 mM HEPES pH 7.5, 100 mM NaCl, 0.03% (wt/vol) DDM. SEC-purified MdtB is incubated with amphipol A8-35 (Anatrace) in the mass ratio 1:5 at 4°C. The free detergents are removed using SM-2 biobeads. Further, amphipol A8-35 exchanged MdtB samples were separated by SEC purification.

### Negative staining TEM sample preparation and imaging

The carbon-coated copper grids from Tedpella (EM grid, 300 mesh; TedPella) were glow discharged for 30 s at 20 mA using the GloQube glow discharge system (Quorum Technologies). The peak fractions of MdtB and MdtF were diluted 40× with the SEC buffer to visualize under room temperature TEM. Four microliters of diluted samples (0.1 mg/mL) were added on the glow-discharged grids and, after 60 s, blotted with a Whatman filter paper. The grid was stained with 1% uranyl acetate (98% uranyl acetate; ACS Reagent, Polysciences Inc. Warrington, PA, USA) followed by blotting and air drying for 2 min. The negatively stained samples were visualized using a 120 kV Talos L120C transmission electron microscope equipped with a bottom-mounted Ceta camera (4,000 × 4,000 pixels). A total of 30 images were collected at a magnification of ×92,000 calibrated with 1.52 Å per pixel.

### Cryo-EM sample preparation

QUANTIFOIL 300 mesh holey carbon grids (R 1.2/1.3) were glow discharged for 70 s at 20 mA using Quorum GlowCube. Three microliters of purified MdtB and MdtF were incubated on the glow-discharged grids, followed by incubating for 10 s before blotting for 8 s at 100% humidity. The grids were quickly plunged into the liquid ethane using a FEI Vitrobot IV plunger (Thermo Fisher Scientific).

### Cryo-EM data acquisition of MdtB and MdtF using 200 kV Talos Arctica

Talos Arctica 200kV cryo-TEM (Thermo Fisher Scientific) is used for cryo-EM data acquisition of MdtB and MdtF to resolve the structure. The instrument is equipped with a K2 direct electron detector (Gatan Inc.). A total of 3,750 movie files were collected for MdtB and 4,000 for MdtF using the LatitudeS automated data acquisition tool (Gatan Inc.) (36). Each movie file contains 20 frames with a total electron dose of 50 e^−^/Å² in the −0.75 to −2.25 μm defocus range. The data were collected at 42,000× magnification with the calibrated pixel size of 1.17 Å for MdtB, and the MdtF data were collected with a nominal magnification of 54,000× at the effective pixel size of 0.92  Å.

### Cryo-EM data processing of MdtB

The micrographs were manually screened in cisTEM software to discard the micrographs with a poor signal-to-noise ratio. The best micrographs after screening using cisTEM were used for the data processing (37). Data were processed using RELION 3.1.2 software (38). Beam-induced motion correction was carried out on the movie files using the MotionCor2 package (39). The contrast transfer function (CTF) estimation of the micrographs was performed using CTFFIND 4.1.13 (40). For generating a 2D class average template for automated particle picking, around 9,214 particles were initially manually picked, and reference-free 2D classification was performed using RELION 3.1.2 (38) to select the best 2D class averages. Around 758,838 particles were auto-picked using the reference of 10 best 2D class averages. Automatically picked particles were extracted with a box size of 300 pixels. Multiple rounds of 2D classification were performed with the extracted particles to clean the particle stack. The good particle set of 352,084 was transferred to cryoSPARC for further data processing (41). *Ab initio* reconstruction was performed with C3 symmetry, and the map was used as a reference with a low-pass filter of 20 Å for further non-uniform refinement using cryoSPARC. After non-uniform refinement with a mask, the final map was sharpened with a −100 B-factor. The final resolution of the refined MdtB map with 252,085 particles with C3 symmetry was determined to be 3.9 Å at 0.143 FSC. The good particle set of 342,913 was used for *ab initio* 3D reconstruction with C1 symmetry. The map was further refined by performing non-uniform refinement with the 20 Å low-pass filtered initial models. The final map was sharpened with a B-factor of −150. The pipeline of EM data processing was shown in the Fig. S5. The structural differences between symmetric and asymmetric reconstruction were analyzed after superposing both the cryo-EM maps in UCSF Chimera and UCSF ChimeraX (42, 43).

### Cryo-EM data acquisition of MdtF using 300 kV Titan Krios

Cryo-EM data were acquired using a 300 kV operated Titan Krios (Thermo Fisher Scientific) microscope equipped with a Falcon direct electron detector (Thermo Fisher Scientific). Micrographs were automatically collected using EPU software (Thermo Fisher Scientific) at a nominal magnification of 75,000× with an effective pixel size of 1.07 Å at the specimen level. These recorded movies have 30 frames, with a total exposure dose of 30.39 e^−^ /Å^2^ at a defocus range of −1.5 to −2.7 µm. A total of 3,000 micrographs were collected for further data processing.

### Cryo-EM data processing of MdtF

The entire single-particle analysis data processing was performed in RELION 3.1.2 (38) and cryoSPARC V4.7 (41). After screening the micrographs using cisTEM, micrographs with poor signal-to-noise ratio were discarded (37). The beam-induced motion correction was carried out on the movie files using MotionCorr2 software (39). The CTF of micrographs was estimated using CTFFIND 4.1.13 (40). Initially, a blob picker was used to pick particles from the subset of micrographs to generate a template for picking. 91,320 particles were extracted with a 300-pixel box size and were sorted by multiple rounds of 2D classification. The final 2D class average was used as a template to pick particles from the full data set. In total, 1,815,916 particles were picked. Multiple rounds of 2D class classification were performed, and, finally, 1,025,190 particles were selected which had a good signal-to-noise ratio. The good particle set was transferred to cryoSPARC for further data processing (41). Ab initio reconstruction was performed with C3 symmetry, and the map was used as a reference with a low-pass filter of 20 Å for further non-uniform refinement. After non-uniform refinement with a mask, the final map was sharpened with a −100 B-factor. The final resolution of the refined MdtF map with C3 was 2.8 Å at 0.143 FSC. The good particle set of 752,103 is used for Ab initio 3D reconstruction with C1 symmetry and split into three. The maps were further refined by performing non-uniform refinement with the 20 Å low pass filtered initial models. The final maps are sharpened with a B-factor of −75. The pipeline of cryo-EM data processing is shown in Fig. S4.

### Model building and analysis

MdtB and MdtF models are generated using the AlphaFold web tool. The generated models are fitted in the map using ChimeraX tool (42). The iMODFIT tool was used to fit the flexible secondary structural regions. The models were real-space refined using the phenix 1.20.1 software, and final refinement was performed using Coot 0.9.8 (44). The models were validated with Phenix 1.20.1 and Coot 0.9.8 software for map-to-model validation, Ramachandran outliers, and clashes. Substrate transport channels and pores were analyzed for MdtF and MdtB using the MOLE online web tool and the CASTp web tool.

## Data Availability

Cryo-EM density maps of the symmetric and asymmetric structure of MdtF (data collected from 300 kV Titan Krios) were deposited in the Electron Microscopy Data Bank (EMDB) under ID EMD-65980 and EMD-63315, respectively. The symmetric map of MdtF (data collected from 200 kV Talos Arctica) is deposited under ID EMD-66716. The symmetric map of MdtB is deposited under EMD-63321. The PDB structure of MdtF is ID 9LYJ, and MdtB is ID 9LYQ.

## SUPPLEMENTAL MATERIAL

Supplemental figures and tables mbio.02684-25-s0001.docx Fig. S1-S9 and Tables S1-S3.

ASM does not own the copyrights to Supplemental Material that may be linked to, or accessed through, an article. The authors have granted ASM a non-exclusive, world-wide license to publish the Supplemental Material files. Please contact the corresponding author directly for reuse.
