# Supplementary material for: Cryo-EM reveals the structural heterogeneity and conformational flexibility of multidrug efflux pumps MdtB and MdtF
Source: mBio. 2025 Dec 10;17(1):e02684-25. doi: 10.1128/mbio.02684-25 (PMC12802153; doi:10.1128/mbio.02684-25)
Supplement: Supplemental figures and tables — Fig. S1-S9 and Tables S1-S3. [file mbio.02684-25-s0001.docx]

**Supplemental Information**

**Cryo-EM reveals the structural heterogeneity and conformational flexibility of multidrug efflux pumps MdtB and MdtF**

Surekha Padmanaban ^1^, Clayton Fernando Rencilin^1^, Rupam Biswas^1,2^, Somnath Dutta^1,*^

^1^Molecular Biophysics Unit, Indian Institute of Science, Bengaluru, India

^2^Department of Physiology and Cell Biology, The Ohio State University, Columbus, United States

*** Corresponding author**: Somnath Dutta (somnath@iisc.ac.in)


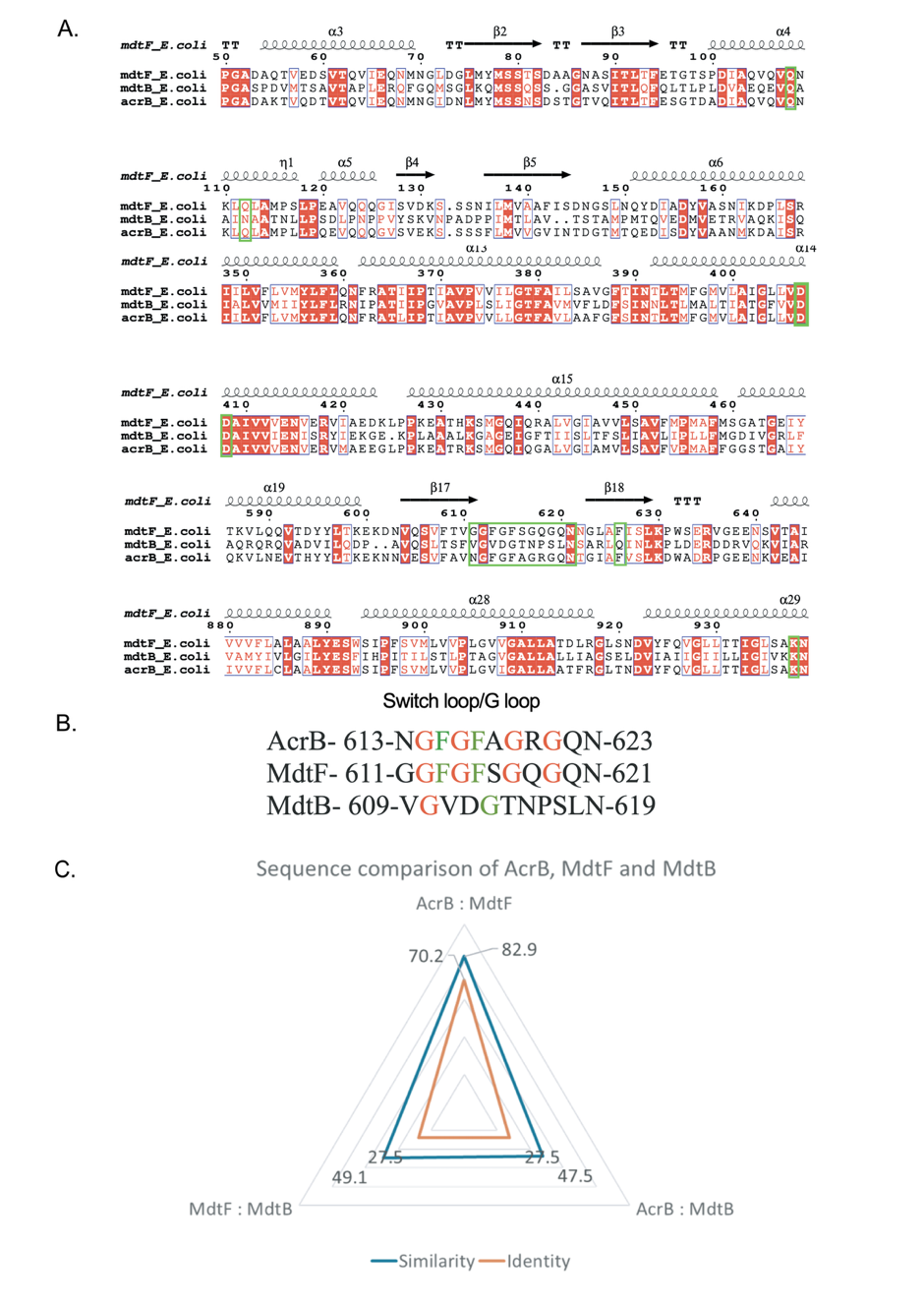


**Supplemental Figure 1:** A. Multiple sequence alignment of RND pumps- MdtB, MdtC, MdtF and AcrB. The conserved amino acid residues in the RND transporter are highlighted in green. Amino acids involved in proton translocation are highlighted in 407, 408 and 938. G-loop region is highlighted from amino acid residues 611- 621 along with the highly conserved F626. B. G-loop sequence of- AcrB, MdtF and MdtB, highlighting the glycine and phenylalanine in the sequence. C. sequence comparison showing the sequence similarity and identity between AcrB, MdtF and MdtB.


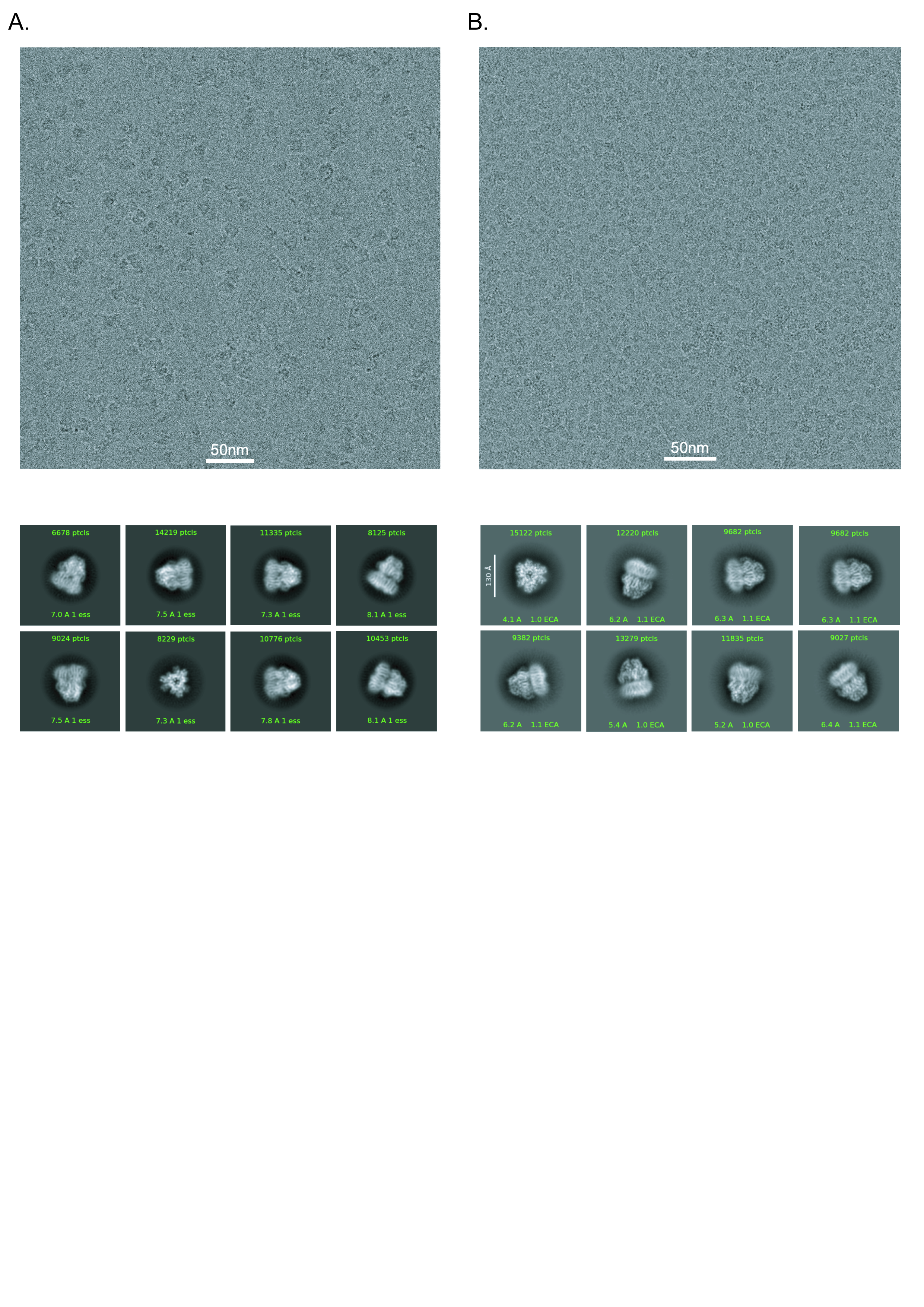


**Supplemental Figure 2:** A. Representative cryo-EM micrograph and 2D class averages of MdtB. B. Cryo-EM micrograph and 2D class averages of MdtF.


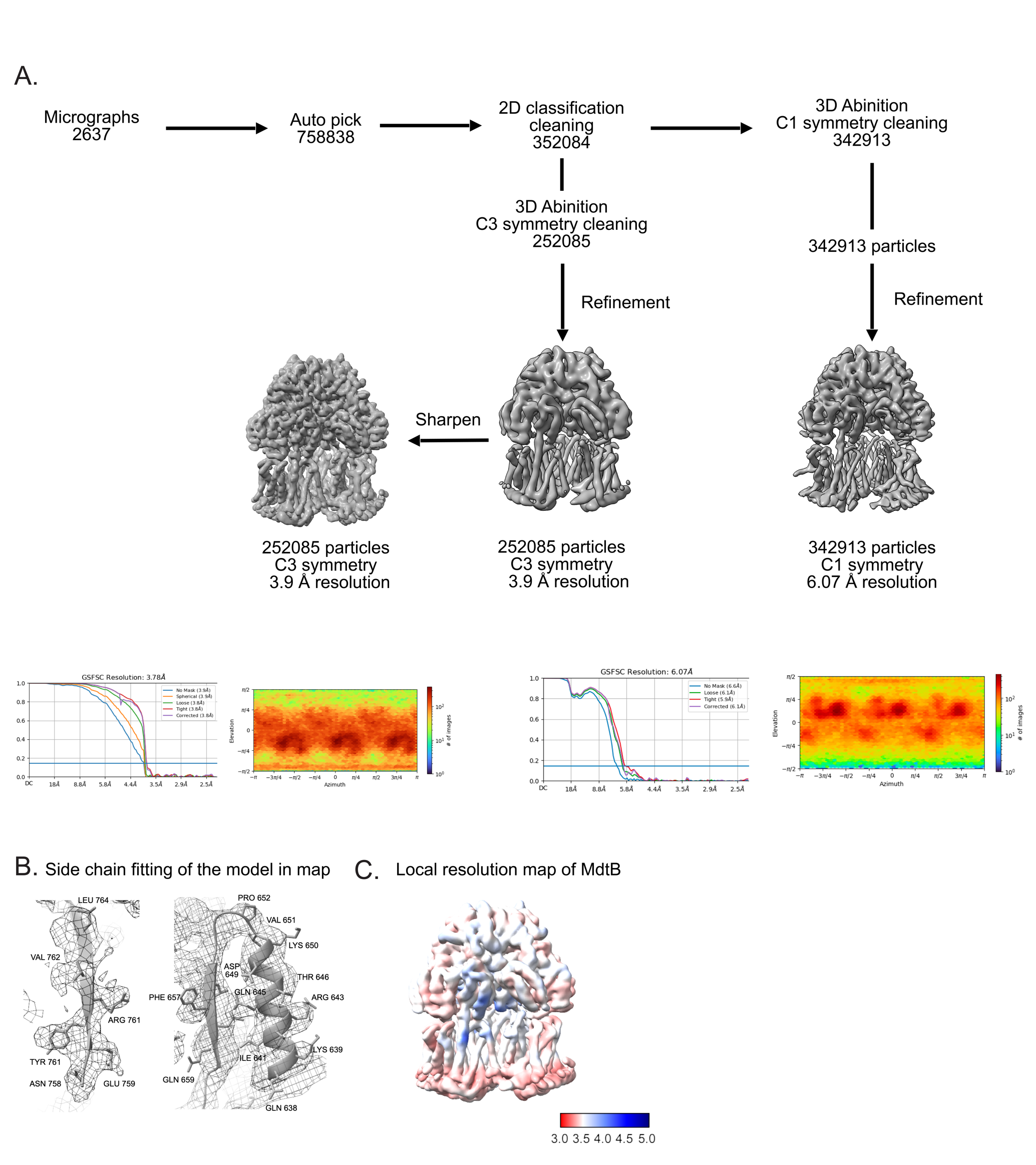


**Supplemental Figure 3:** A. Cryo-EM data processing workflow of MdtB. B. MdtB model fitted in the cryo-EM density map of MdtB. B. local resolution map of reconstructed homotrimeric MdtB.


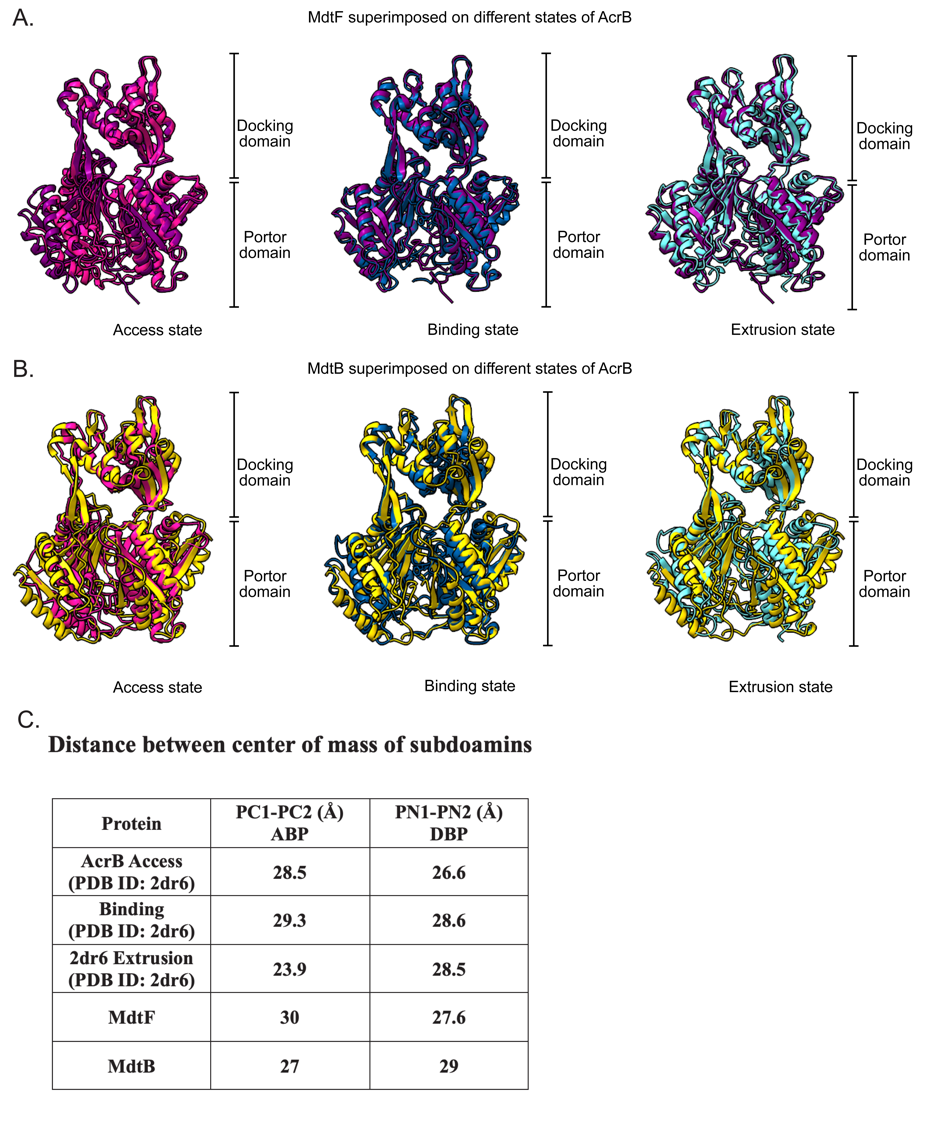
**Supplemental Figure 4:** Comparison of MdtF and MdtB with different states of RND pump. A. Comparison of access state, binding state, and extrusion state with MdtF. B. Comparison of access state, binding state, and extrusion state with MdtB. C. Distance between the center of mass of subdomains PC1-PC2 and PN1-PN2 are measured for MdtF, MdtB and AcrB (PDB ID: 2dr6) Access state, binding state and extrusion state. Colour code: Yellow – MdtB; Pink - Access state; Blue - Binding state; Cyan - Extrusion state


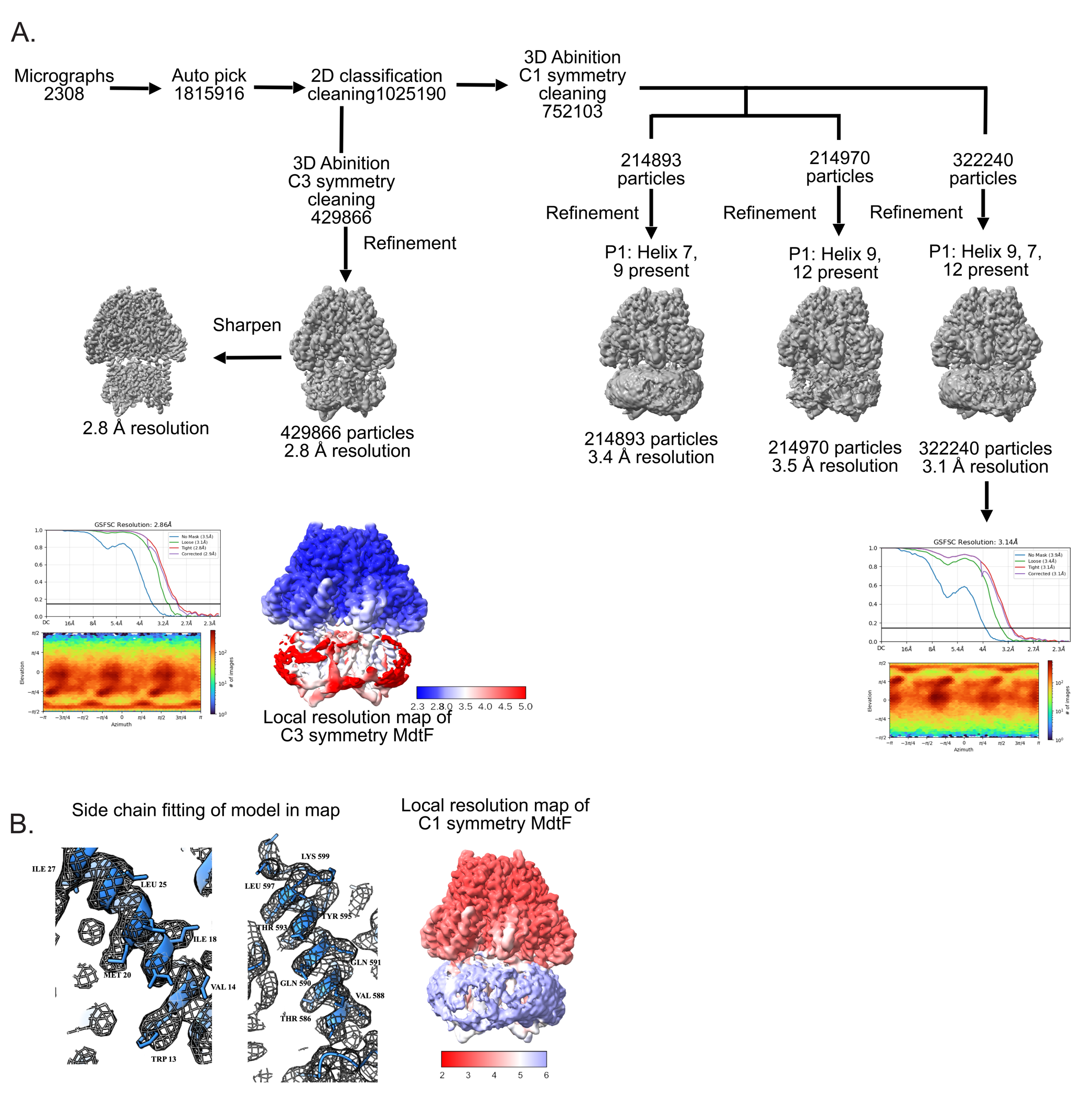


**Supplemental Figure 5:** A. Cryo-EM data processing workflow of MdtF. B. MdtF model fitted in the cryo-EM density map of MdtF. B. local resolution map of reconstructed MdtF.


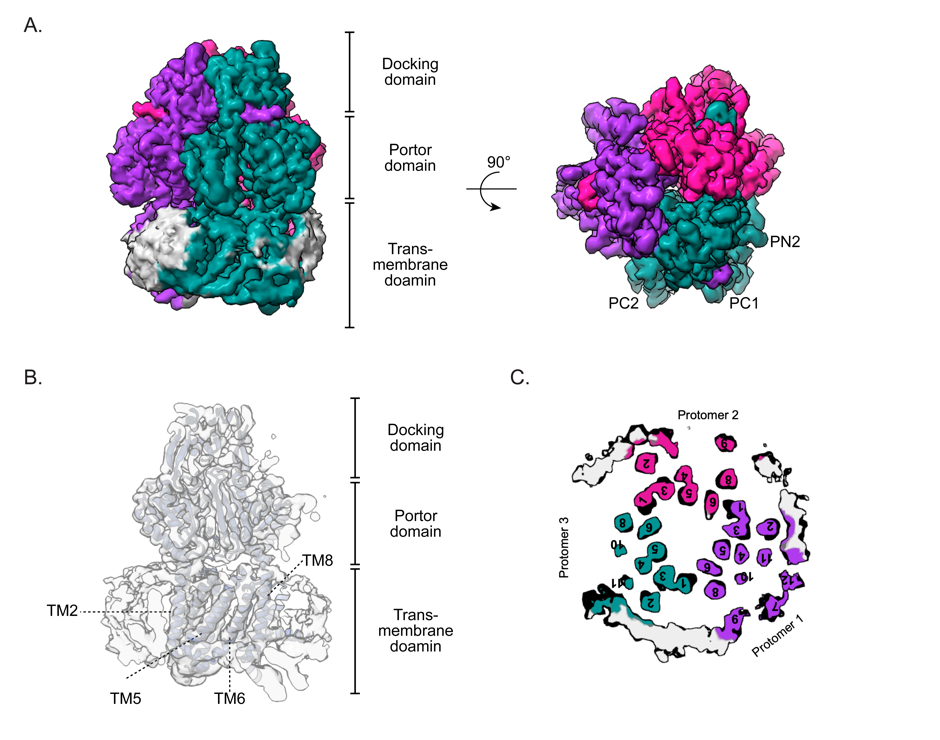
**Supplemental Figure 6:** Asymmetric reconstruction of MdtF, Class1. A. Protomer 1 with all the resolved transmembrane helices solved the structure of the peripheral helices in MdtF. B. MdtF model fit in the map. The posterior view is displayed to represent the core helix fitting. C. Top view of the resolved helix of protomer 1.

**
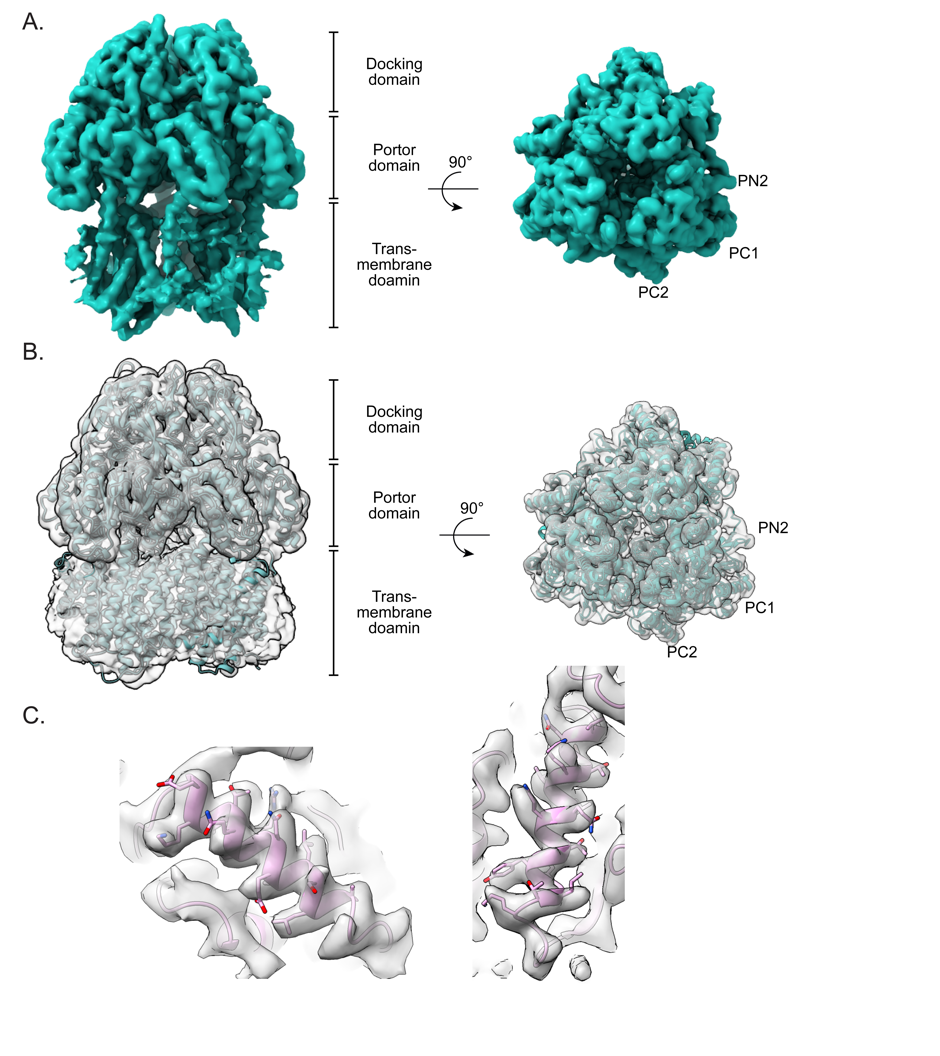
**

**Supplemental Figure 7:** MdtF structure solved with 200 kV Talos Arctica: A, MdtF cryo-EM map resolved at 3.3 Å resolution. B, MdtF model fitting in cryo-EM map. C, Sidechain fitting of the helix.


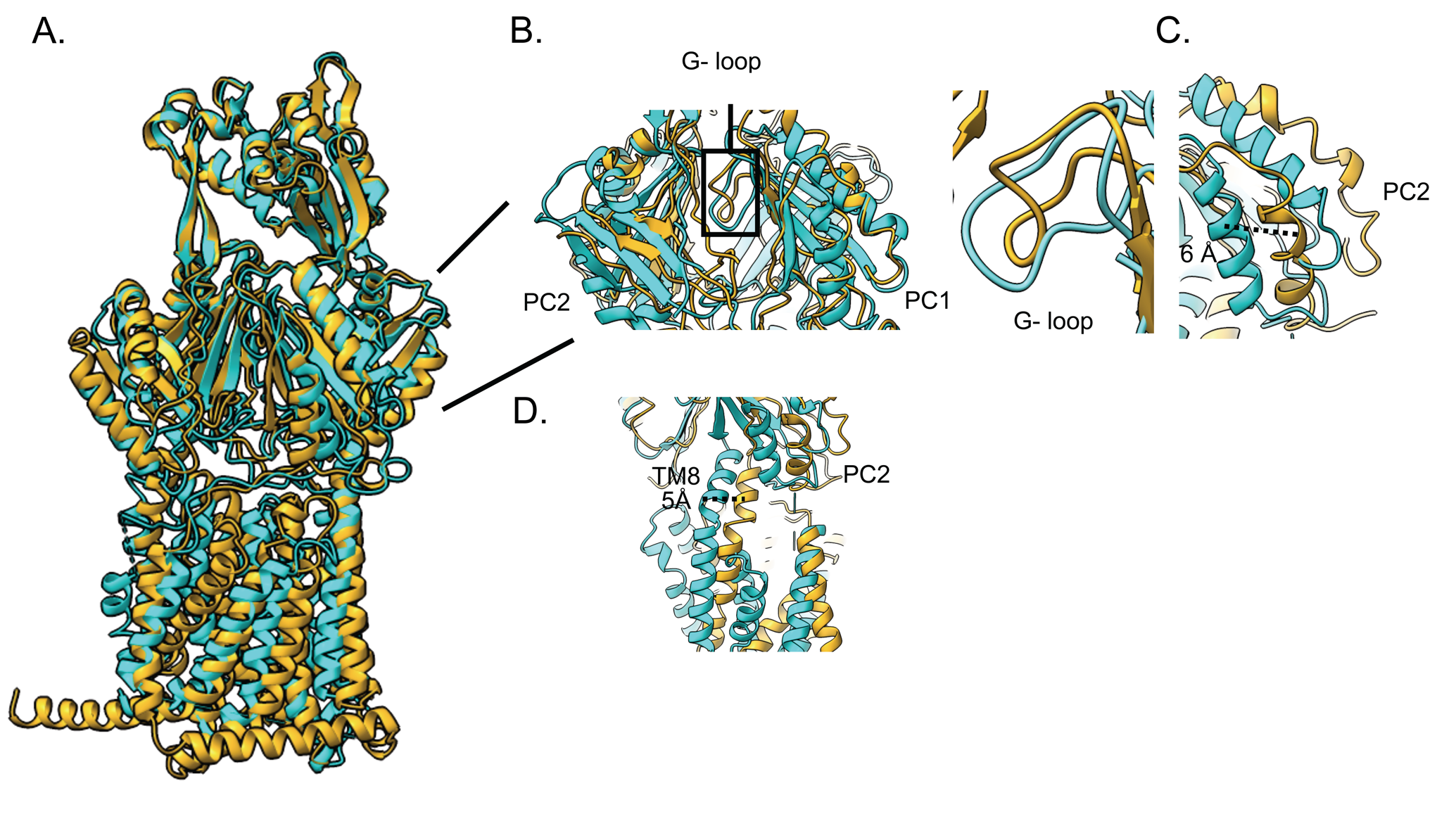


**Supplemental Figure 8:** Comparison of MdtF with MdtB. A. MdtF superimposed on MdtB. B, G loop of MdtF and MdtB. C. PC1 movement comparison between both the proteins. D. TM8 and PC2 comparison between MdtF and MdtB. Cyan colour represents MdtF and yellow colour model represent MdtB


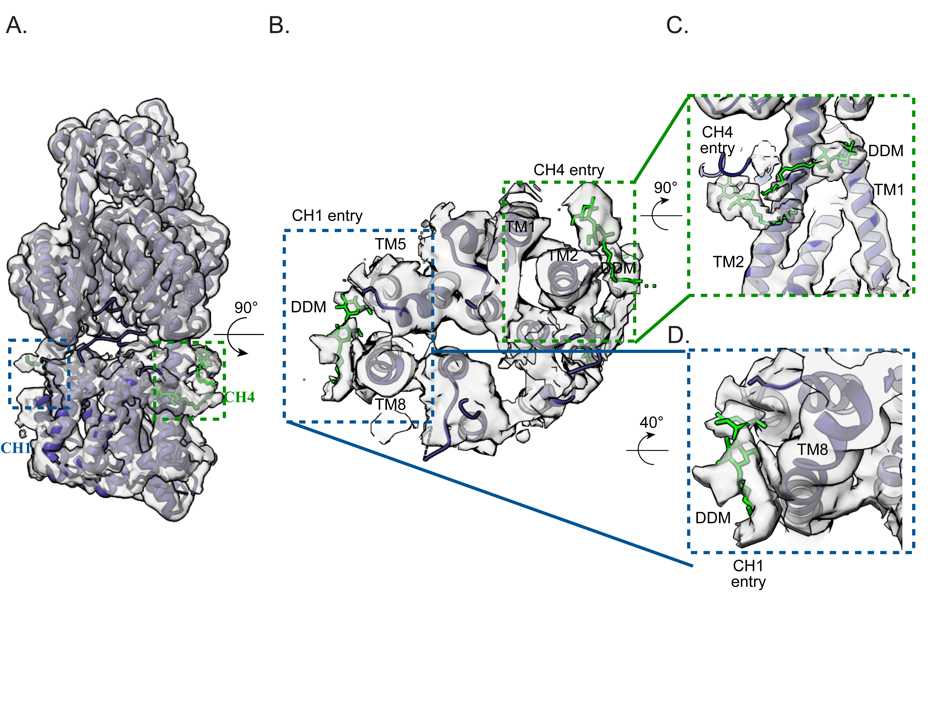


**Supplemental Figure 9:** A. Side view of MdtF and DDM fitted in the map showing the CH1 and CH4 entry labelled in blue and green, respectively. B. Top view showing the MdtF and DDM fitted in the cryo-EM density map with the CH1 and CH4 entry labelled in blue and green, respectively. DDM fitted in the MdtF density map. C. Side view of channel 4 (CH4) showing the fitted DDM in MdtF map density. D. Tilted view (45º) of channel 1 (CH1) showing the fitted DDM in MdtF density map.

**Table S1**: **Cryo-EM Data collection, image processing and refinement for MdtF collected from 300-kV operated Titan Krios (Thermo Fisher Scientific)**

| **Data Collection and Processing** | **MdtF-300kV** (asymmetric) | **MdtF-300kV** (symmetric) |
| --- | --- | --- |
| Magnification | 75,000x | 75,000x |
| Voltage | 300kV | 300kV |
| Electron exposure (e^-^/Å^2^) | 30.39 e^-^ /Å^2^ | 30.39 e^-^ /Å^2^ |
| Defocus range (µm) | -1.5 μm to -2.7 μm | -1.5 μm to -2.7 μm |
| Pixel size (Å) | 1.07 Å | 1.07 Å |
| Symmetry Imposed | C1 | C3 |
| Number of Particles | 322240 | 429866 |
| Map Resolution (Å) | 3.1Å | 2.8Å |
| FSC threshold | 0.143 | 0.143 |
| Map Sharpening B Factor (Å^2^) | -75 | -100 |
| Number of Movies | 3000 | 3000 |

**Table S2: Cryo-EM Data collection, image processing and refinement for MdtB and MdtF datasets collected form Talos Arctica 200kV cryo-TEM (Thermo Fisher Scientific):**

| **Data Collection and Processing** | **MdtB – 200kV** | **MdtF – 200kV** |
| --- | --- | --- |
| Magnification | 42000x | 54000x |
| Voltage | 200kV | 200kV |
| Electron exposure (e^-^/Å^2^) | 50 e-/Å² | 50 e-/Å² |
| Defocus range (µm) | -0.75 μm to -2.25µm | -0.75 μm to -2.25µm |
| Pixel size (Å) | 1.17 Å | 0.92 Å |
| Symmetry Imposed | C3 | C3 |
| Number of Particles | 252085 | 569547 |
| Map Resolution (Å) | 3.9 Å | 3.3 Å |
| FSC threshold | 0.143 | 0.143 |
| Map Sharpening B Factor (Å^2^) | -100 Å | -70 Å |
| Number of Movies | 3750 | 4000 |

**Table S3: Refinement statistics for MdtB and MdtF:**

| **Validation** | **MdtB** | **MdtF** |
| --- | --- | --- |
| MolProbity Score | 2.29 | 1.89 |
| Clash Score | 17.38 | 17.94 |
| Ramachandran Plot |  |  |
| Favored (%) | 97.33 | 97.28 |
| Allowed (%) | 2.67 | 2.51 |
| outliers (%) | 0 | 0.21 |
